# Nano-metals forming bacteria in Egypt. I. Synthesis, characterization and effect on some phytopathogenic bacteria in vitro

**DOI:** 10.1038/s41598-021-92171-6

**Published:** 2021-06-18

**Authors:** Sahar Abd El-Fatah Zaki, Ayman Kamal, Nader A. Ashmawy, Alia A. Shoeib

**Affiliations:** 1 Environmental Biotechnology Department, Genetic Engineering and Biotechnology Research Institute, City of Scientific Research and Technological Applications, Alexandria, Egypt; 2 Plant Pathology Department, Faculty of Agriculture, Alexandria University, Alexandria, Egypt

**Keywords:** Microbiology, Plant sciences, Nanoscience and technology

## Abstract

Bacterial metal reducers were isolated from water samples collected from harsh condition locations in Egypt. Four selected isolates were identified as *Enterococcus thailandicus, Pseudomonas putida, Marinobacter hydrocarbonoclasticus*, and *P. geniculata* for Copper (Cu), Iron (Fe)*,* Cobalt (Co) and Zinc (Zn) Nanoparticles (NPs) production sequentially. Nitrate reductase enzyme was assayed for bacterial isolates which demonstrated that *P. putida*, and *M. hydrocarbonoclasticus* have the maximum enzyme production*.* The produced NPs were characterized by using XRD, TEM, UV–VIS spectroscopy. Magnetic properties for all selected metals NPs were measured using Vibrating Sample Magnetometer (VSM) and demonstrated that FeNPs recorded the highest magnetization value. The antibacterial activity of selected metals NPs was tested against some phytopathogenic bacteria causing the following diseases: soft rot (*Pectobacterium carotovorum, Enterobacter cloacae*)*,* blackleg (*Pectobacterium atrosepticum* and *Dickeya solani*), brown rot (*Ralstonia solanacearum*), fire blight (*Erwinia amylovora*) and crown gall (*Agrobacterium tumefaciens*). All metals NPs showed an antagonistic effect against the tested isolates, particularly, FeNPs showed the highest antibacterial activity followed by CuNPs, and ZnNPs. Due to the small size, high reactivity, and large surface area of biologically synthesized NPs, they are used as a good disinfector, and can be considered as a new and alternative approach to traditional disease management methods.

## Introduction

Plants are exposed to infections by a large spectrum of over 200 pathogenic bacterial species that cause diseases that can lead to damage and death^1^**.** There are many ways to control bacterial plant diseases such as antibiotics; due to the natural development of bacterial resistance^2^ often antibiotics are ineffective in disease management^3^. Using chemical pesticides cause a hazardous effect on the environment, animals, and human health^4^. Nanotechnology as a new approach was applied to the development of novel antibacterial agents for the management of phytopathogenic bacteria affecting crops. The reduction of macro materials into Nanoscale particles (1–100 nm) gives hope to new characteristics and the material behaves differently^5^.

Different chemicals^6^, physical^7^**,** and biological^8,9^ processes are currently widely used to synthesize metallic nanoparticles (NPs) for use as antibacterial agents. Many biological systems, can convert inorganic metal ions into metal NPs via the reductive capacities of the bioproduct of these organisms. The low cost of cultivation, low energy requirements, short production time, safety, and the ability to up production volumes and eco-compatibility make biological synthesis an attractive platform for nanoparticle synthesis alternative to physical and chemical preparation^10^.

The physicochemical properties of NPs are important for their behaviour, bio-distribution, safety, and efficacy. Therefore, the characterization of NPs is important to evaluate the functional aspects of the synthesized particles. Characterization is performed using a variety of analytical techniques, including UV- VIS spectroscopy^11^, X-ray diffractometry^12^, Raman scattering^13,14^, Energy-Dispersive X-Ray^15^, Transmission electron microscopy^16^, vibrating sample magnetometer^17^, Particle size analysis^18^, and inductively coupled plasma-optical emission spectrometry^19^.

The objectives of this study were a biological synthesis of different types of metallic NPs using eco-friendly bacterial isolates obtained from the Egyptian ecosystem, production, and characterization of these metals NPs from bacterial isolates, as well as, studying their effects on some phytopathogenic bacteria in vitro.

## Results

### Chemical analysis of water samples

The experimental data on the chemical parameters of the collected water samples are presented in Table 1. Data demonstrated that all water samples contained inorganic heavy metals pollutants such as Fe^3+^, Zn^2+^, Cu^2+^, and highly toxic heavy metals like cadmium (Cd^2+^) and Pb^2+^ as well. Sample A1 contained 19 mg/L lead (Pb^2+^) and 25 mg/L Cd^2+^, sample A2 contained 5.1 mg/L Cu^2+^ and 3.4 mg/L Fe^3+^, and also sample A5, A6, A7, and A8 contained traces of Pb^2+^ and Cd^2+^. Sample A5 and A6 showed the highest pH values 8.6 and 8.1 respectively. Besides, sample A1 recorded the highest nitrate and salinity concentration 87 mg/L, 95.1 psu.

**Table 1:** Chemical analyses of water samples.

Parameter	pH	No^2−^ mg/L	No^3−^ mg/L	Salinity (psu)	Fe^3+^ mg/L	Zn^2+^ mg/L	Cu^2+^ mg/L	Pb^2+^ mg/L	Cd^2+^ mg/L
Sample									
A 1	6.59	62	87	95.1	13	1033	282	19	25
A 2	7.4	0.04	0	56.6	3.4	1.2	5.1	0.094	0.036
A 3	7.5	1.8	0	41.2	0.07	0.4	0.1	0.006	0.002
A 4	7.6	0.78	0	42.1	0.01	0.3	0.2	0.01	0.0
A 5	8.6	0.12	0	46.7	0.4	0	2.8	0.006	0.81
A 6	8.1	2.1	2.6	22.6	1.3	0.9	1.8	0.15	0.07
A 7	7.9	1.8	1.0	34.2	0.02	0.07	0.1	0.002	0.03
A 8	7.9	0.621	1.6	48.3	0.01	0.08	0.07	0.12	0.01

### Isolation of nano-metals forming bacteria

**Figure 1:**
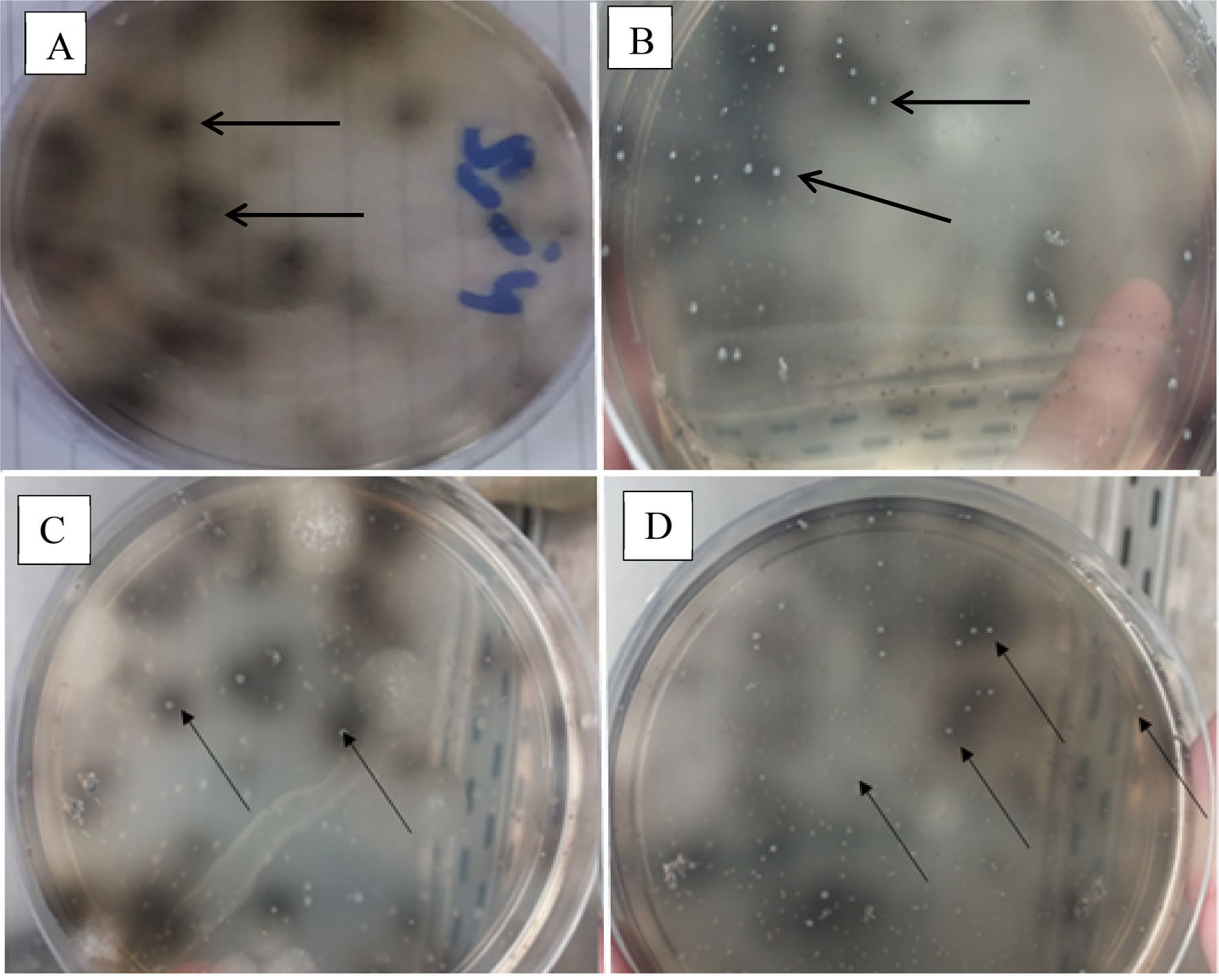
Selective isolation of metals reducing bacteria. (**A**) sample A1 cultured on Iron nitrate, (**B**) sample A4 cultured on Copper nitrate (**C**) sample A7 cultured on Cobalt nitrate (**D**) sample A8 cultured on Zinc nitrate.

Table 2 shows visual observation and selective isolation of Nano metals forming bacteria on LB medium supplemented with metals nitrates (Fe^3+^, Cu^2+^, Co^2+^or Zn^2+^). After 6 days incubation, results indicated that samples A1, A4, A7, and A8 contained metal-reducing bacteria, as shown in Fig. 1. Reduction of metals by bacteria resulted in dark brown colonies (isolate T65 from sample A1) or dark zone around bacterial growth, which was recorded in 3 isolates: E12, M69, and G80 from 3 samples A4, A7, and A8 respectively.

**Table 2:** Isolation of metals reducing bacteria from water samples.

Metals salts	L.B medium + Fe^2+^ nitrate	L.B medium + Cu^2+^ nitrate	L.B medium + Co^2+^ nitrate	L.B medium + Zn^2+^ nitrate	Isolate code
Sample					
A 1	Color change	ND	ND	ND	T65
A 2	ND *	ND	ND	ND	–
A 3	ND	ND	ND	ND	–
A 4	ND	Color change	ND	ND	E12
A 5	ND	ND	ND	ND	–
A 6	ND	ND	ND	ND	–
A 7	ND	ND	Color change	ND	M69
A 8	ND	ND	ND	Color change	G80

*ND Not detected.

### Determination of nitrate reductase

**Figure 2:**
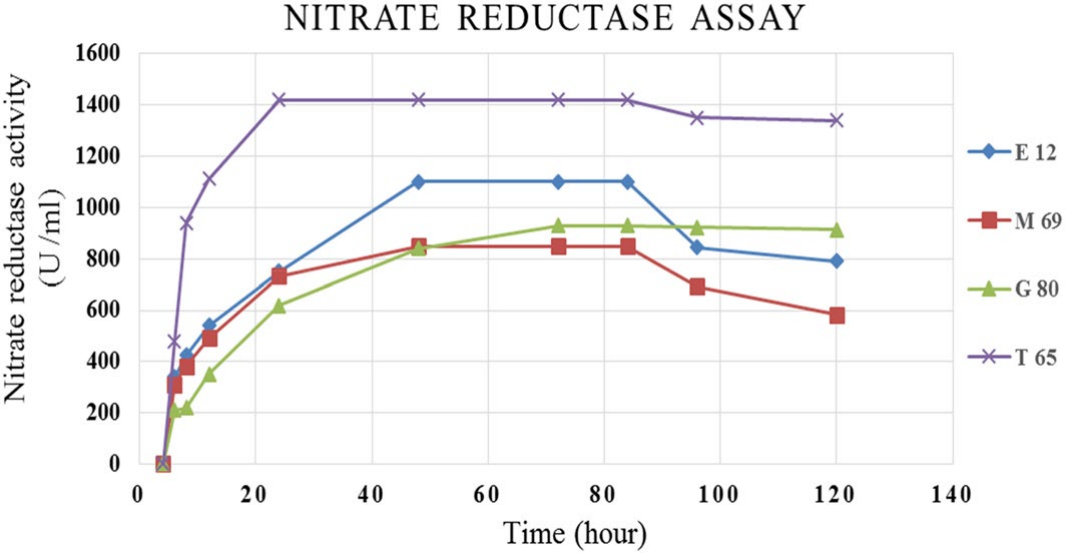
Time course of enzyme production around 120-h incubation of selected isolates.

Nitrate reductase enzyme was produced by selected isolates T65, E12, M69, and G80 grown on Fe^3+^, Cu^2+^, Co^2+^, and Zn^2+^ nitrate respectively. Figure 2, demonstrated that maximum enzyme production was around 20 to 80-h of incubation and the maximum production was 1400 U/mL in the case of isolate T56 after 24-h incubation and the minimum production was 850 U/mL in the case of isolate M69 after 84-h incubation.

### Morphological, physiological, and biochemical tests of NPs forming bacteria

Data on morphological, physiological, and biochemical characterization of selected bacterial isolates T65, E12, M69, and G80 (S1) showed that isolates belonged to *Pseudomonas*, *Enterococcus*, and *Marinobacter* spp.

### Molecular identification of NPs forming bacteria using 16S rRNA gene sequences

The resulting partial DNA sequences of the 4 bacterial isolates were analyzed using the BLAST tool in NCBI (National Center for Biotechnology Information) www.ncbi.nlm.nih.gov.

Obtained data analyses revealed that inferred 16S rRNA sequences of isolates showed similarity to *P. putida, E. thailandicus, M. hydrocarbonoclasticus*, *and P. geniculata*. The bacterial isolates were illustrated with the Gene Bank accession numbers in S2.

### Alignment and phylogenetic analysis

**Figure 3:**
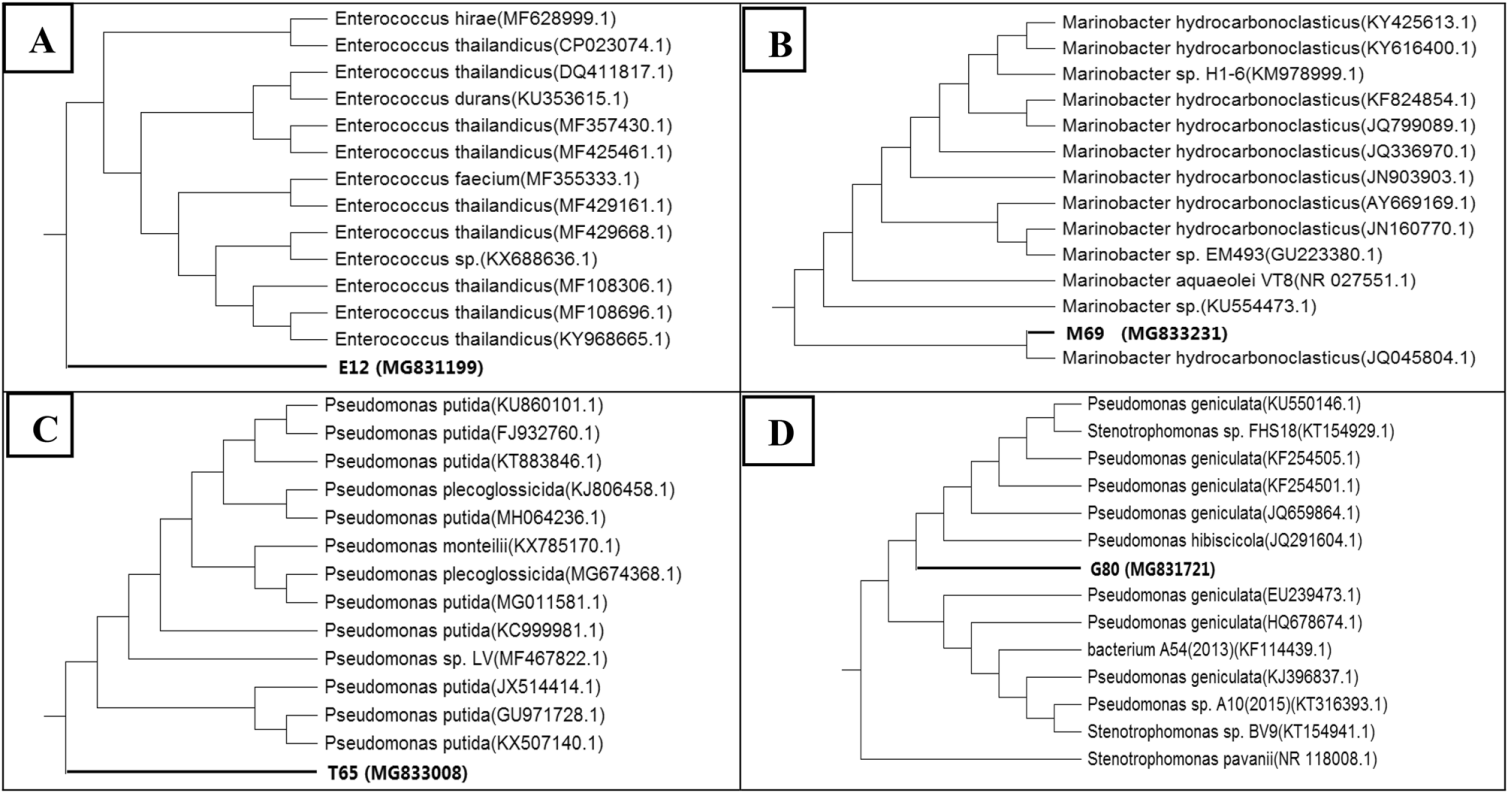
Phylogenetic analyses of NPs forming bacteria isolate E12 (**A**), isolate M69 (**B**), T65 (**C**), and isolate G80 (**D**) based on 16S rRNA sequence analysis, using MEGA version 6.1 from CLUSTALW alignment, at the NCBI site (http://www.ncbi.nlm.nih.gov).

Based on nucleotide homology and phylogenetic analysis, the isolate E12 showed the highest similarity (98.0%) with *E. thailandicus* KY968665.1, isolate M69 showed the highest similarity (99.0%) with *M. hydrocarbonoclasticus* JQ045804.1, as well isolate T65 showed the highest similarity (98.0%) with *P. putida* KU860101.1, also, the isolate G80 showed the highest similarity (99.0%) with *P. geniculata* KU550146. The phylogenetic trees of selected isolates were related to isolates from the gene bank (Fig. 3).

### Production of metals NPs

The reduction of metals ions to metals NPs could be optically appreciated after incubation of the bacterial culture by color changes of the medium from yellow to dark brown/black as illustrated in S**3**.

### Characterization of metals NPs

#### UV–VIS spectroscopic analysis

**Figure 4:**
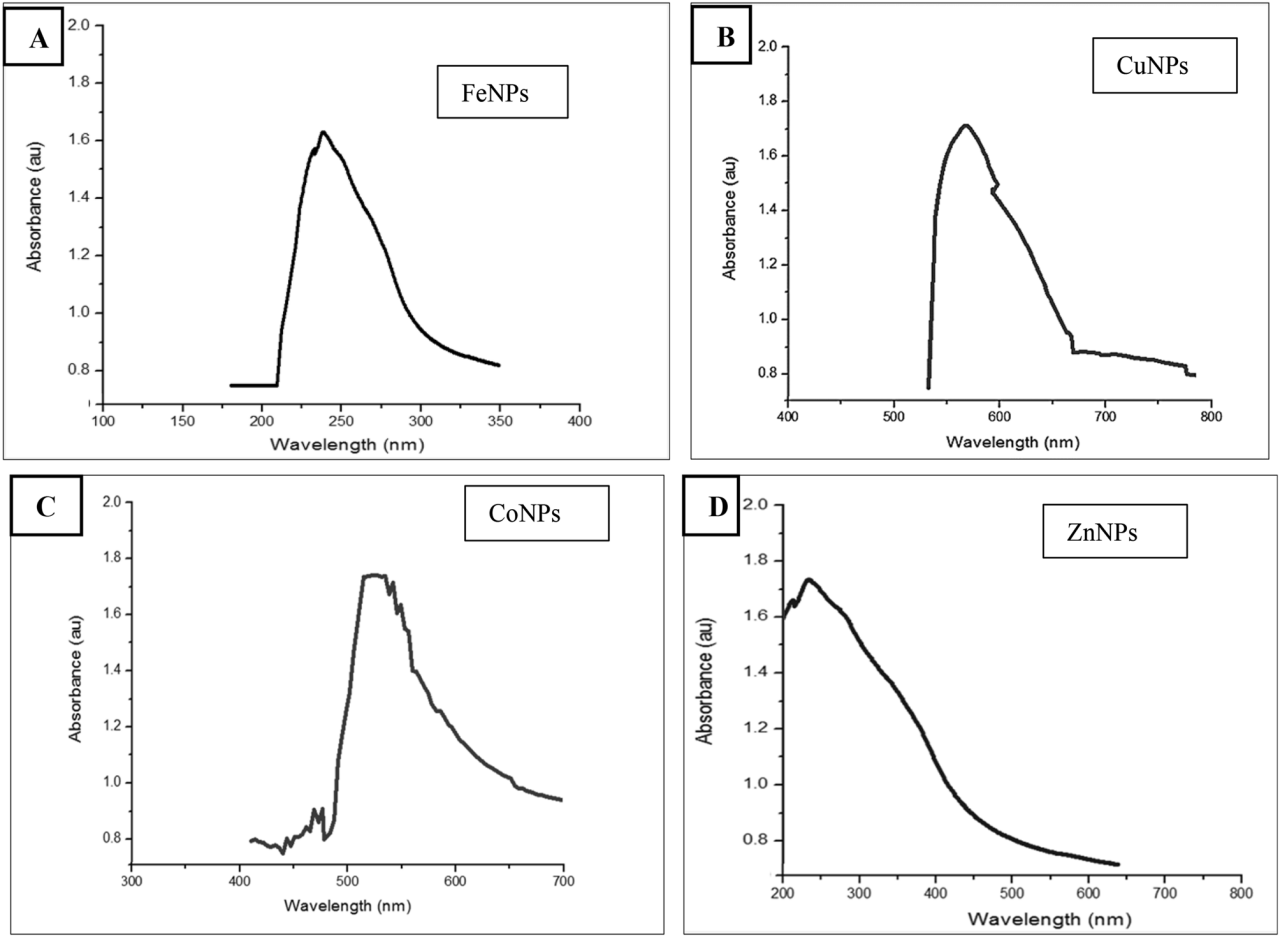
UV–VIS. spectra of FeNPs (**A**), CuNPs (**B**), CoNPs (**C**) and ZnNPs (**D**).

The UV–VIS spectroscopic analysis of synthesized metals NPs was shown in (Fig. 4). UV–VIS spectroscopic analysis of FeNPs and ZnNPs (Fig. 4A,D) showed the absorption maximum peak at 233 nm and around 230–250 nm respectively. The maximum absorption peaks of CuNPs and CoNPs appeared at around 569 nm (Fig. 4B), and 530 nm (Fig. 4C) consecutively.

#### Transmission electron microscope (TEM) analysis

**Figure 5:**
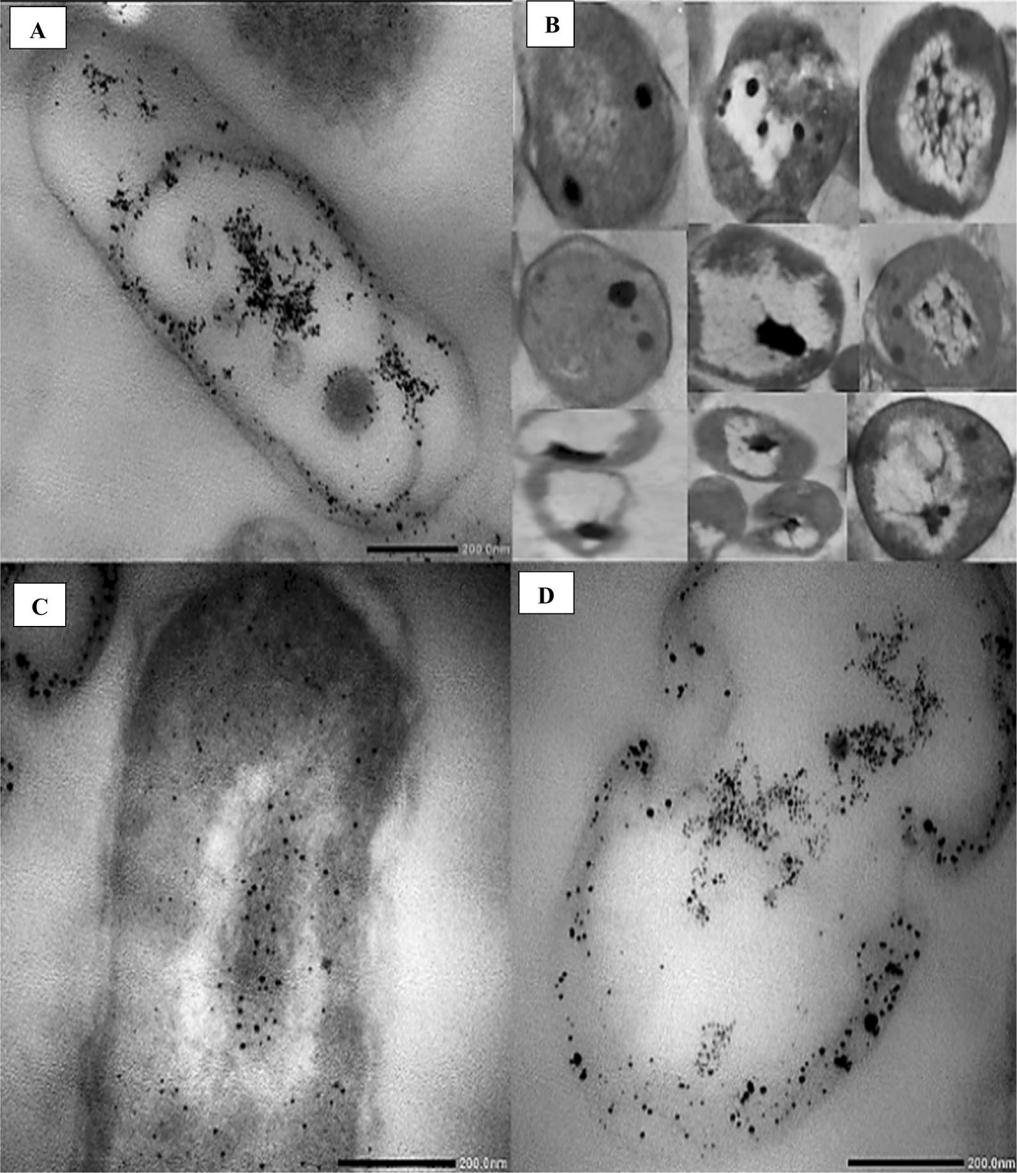
Intracellular biosynthesis of metals NPs, FeNPs in *Pseudomonas putida* (**A**), CuNPs in *Enterococcus thailandicus* (**B**), CoNPs in *Marinobacter hydrocarbonoclasticus* (**C**), and ZnNPs in *P. geniculata* isolate (**D**).

The Ultrastructure study of the intracellular biosynthesis of metals NPs was examined using TEM. Particles with higher electron density appear darker in the TEM negative film. Figure 5 shows that the synthesis of FeNPs and ZnNPs is in both periplasmic space and the cytoplasm of the bacterial cell of *P. putida* (Fig. 5A) and *P. geniculata* (Fig. 5D) respectively, while synthesis of CuNPs and CoNPs, was in the cytoplasm of cells of *E. thailandicus* (Fig. 5B) and *M. hydrocarbonoclasticus* (Fig. 5C) respectively.

**Figure 6:**
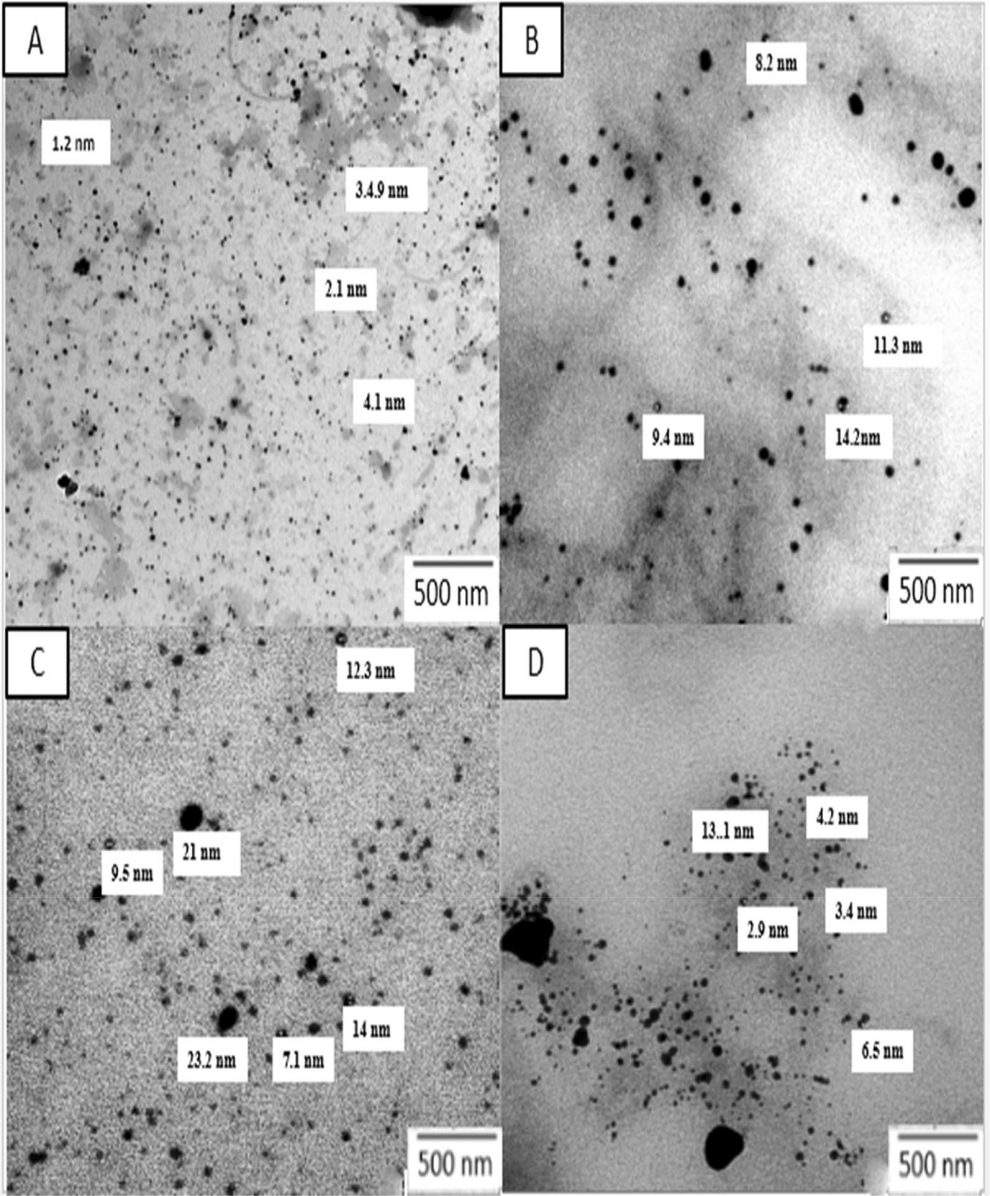
Transmission Electron Microscope (TEM) analysis of metals NPs, FeNPs (**A**), CuNPs (**B**), CoNPs (**C**) and ZnNPs (**D**).

The spherical morphology and size of metals NPs are shown in Fig. 6. Synthesized FeNPs, CuNPs, CoNPs, and ZnNPs were in the range of 1–4, 8–14, 9–20, and 4–13 nm, respectively.

#### X-ray powder diffraction (XRD) analysis

**Figure 7:**
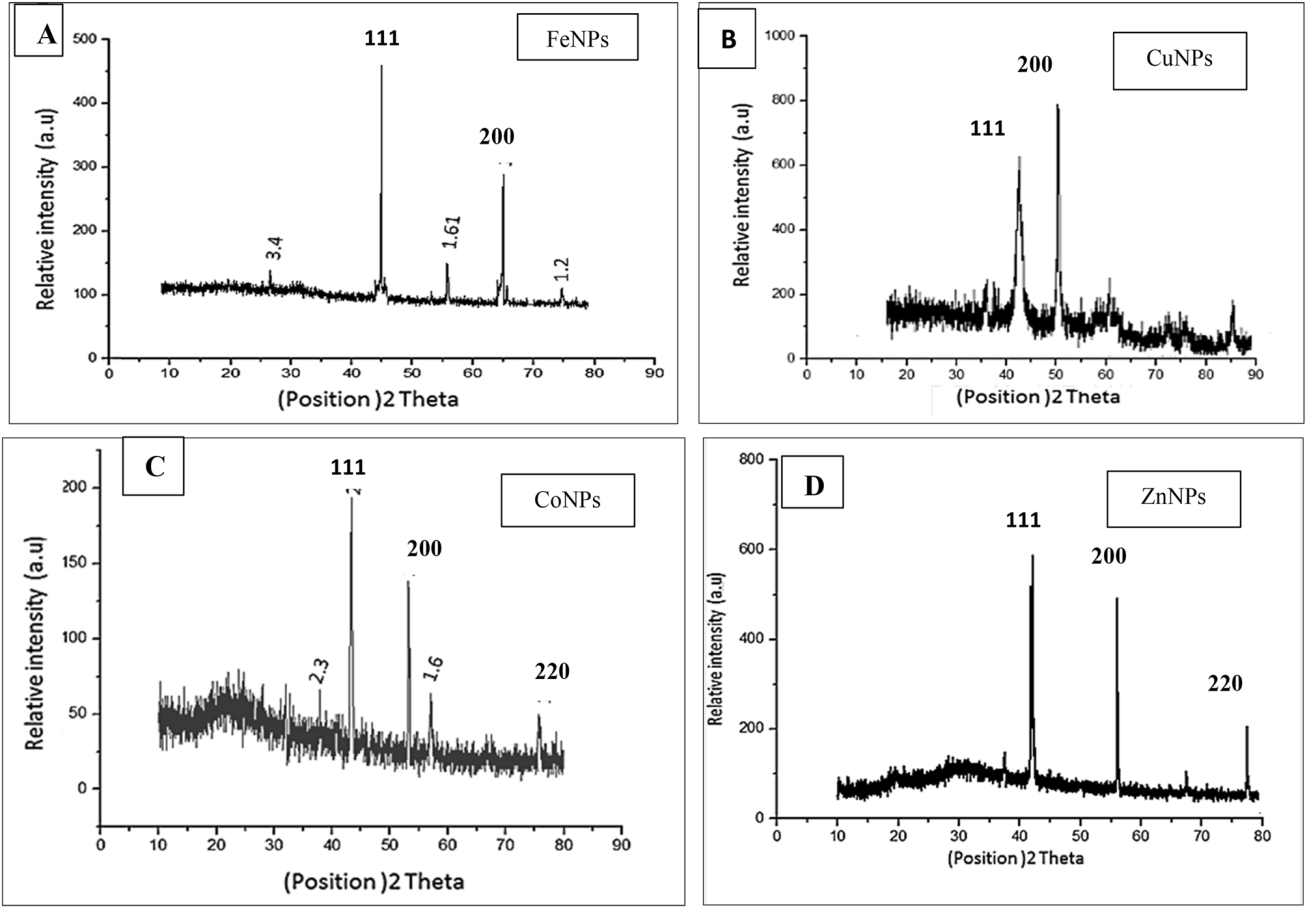
X-ray powder diffraction pattern of FeNPs (**A**), CuNPs (**B**), CoNPs (**C**) and ZnNPs (**D**).

The crystalline nature, quality, and crystallographic identity of the examined NPs in addition to the phase purity were determined by the XRD spectrum over a wide range of Bragg angles 10° ≤ 2θ ≤ 80. XRD pattern was characterized by the interplanar d-spacing/2θ degree and the different intensities of the strongest peaks. The X-ray diffractograms of metals NPs synthesized by the selected bacterial isolates are illustrated in Fig. 7. The XRD pattern of FeNPs in Fig. 7A reveales that a peak of the highest intensity occurs at 45.50°. Tow main characteristic diffraction peaks for CuNPs were observed (Fig. 7B) at around 2θ = 42°, 50°. The XRD pattern (Fig. 7C) of CoNPs shows the diffraction peaks at 43.2°, 54.5°, and 77.9°, also XRD pattern in Fig. 7D of ZnNPs exhibits diffraction peaks at about 2θ = of 43°, 56°, and 79.83°.

#### Raman spectroscopy analysis

**Figure 8:**
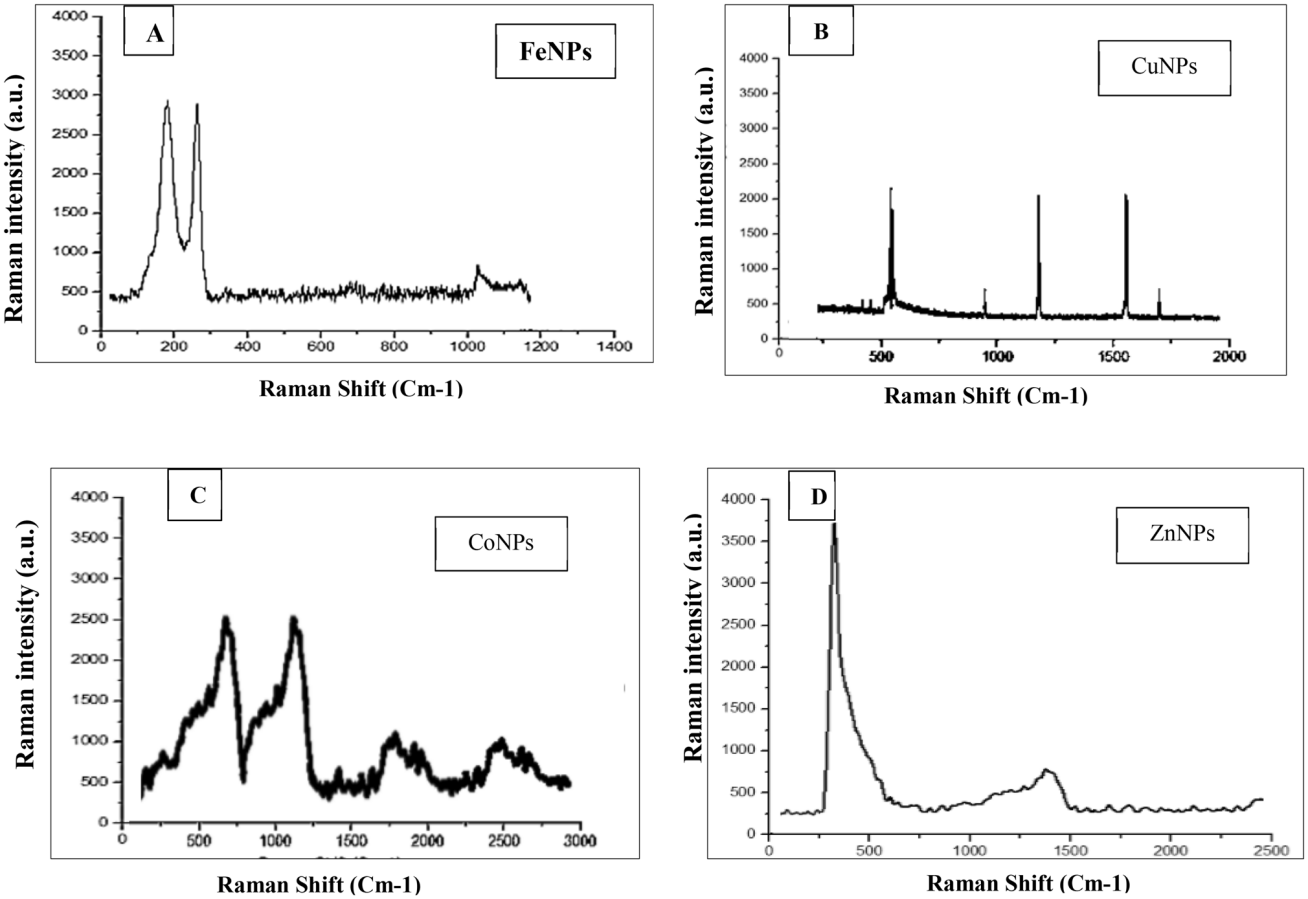
Raman spectra of FeNPs (**A**), CuNPs (**B**), CoNPs (**C**) and ZnNPs (**D**).

Raman spectroscopy allows the characterization of many types of samples without any specific requirement preparation. NPs Raman spectra of metallic NPs are shown in Fig. 8. Three peaks were observed around 200, 290, and 1100 cm^−1^ for FeNPs (Fig. 8A). CuNPs absorptions peaks were around at 500 cm^−1^, 1250 cm^−1^, and 1500 cm^−1^ (Fig. 8B). In addition, CoNPs (Fig. 8C) absorption peaks were around 500 cm^−1^ and 1000 cm^−1^, and (Fig. 8D), around 300–500 cm^−1^ and 1400 cm^−1^ in the case of ZnNPs.

#### Particle size analysis.

**Figure 9:**
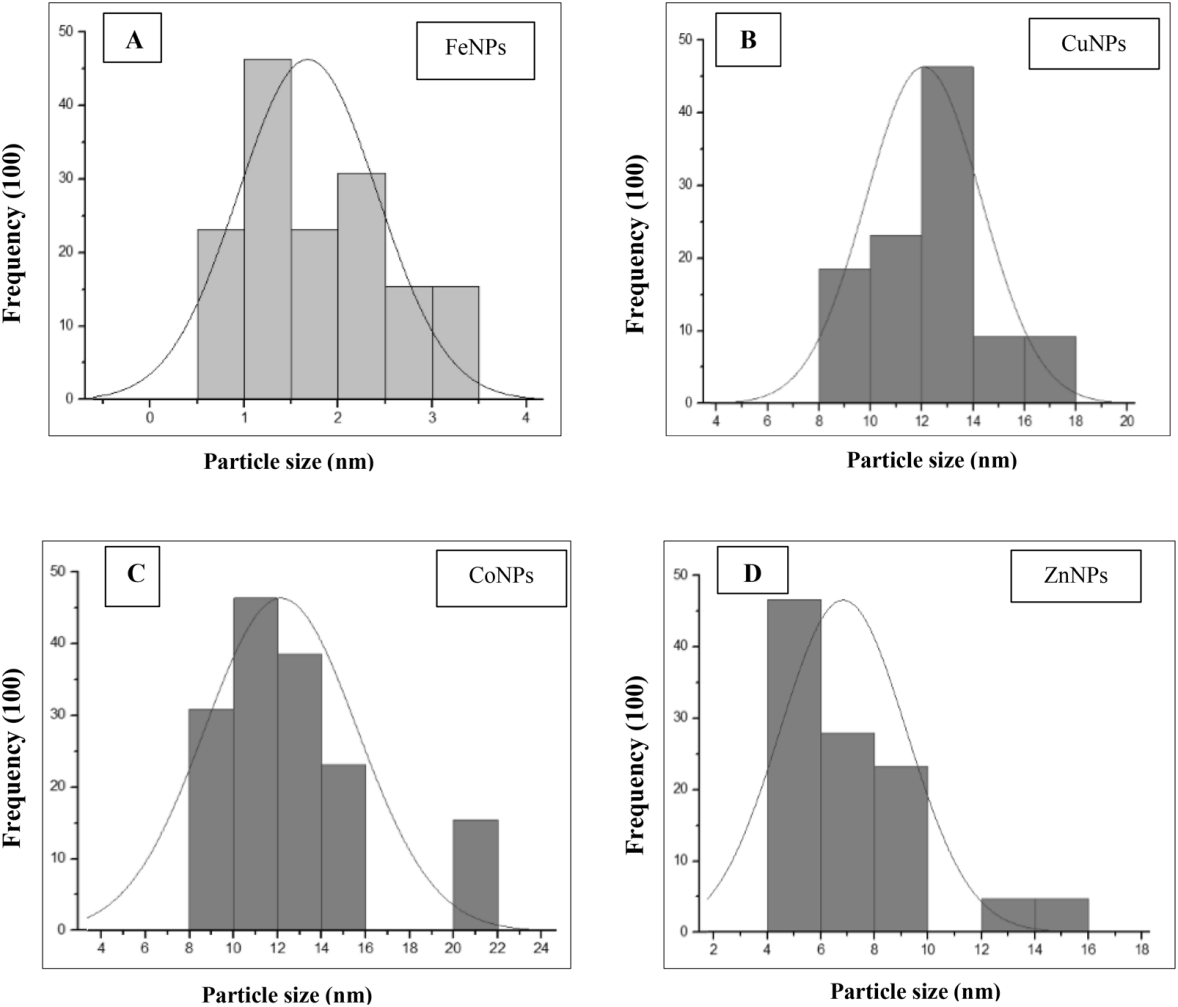
Particle size distribution histogram of FeNPs (**A**), CuNPs (**B**), CoNPs (**C**) and ZnNPs (**D**).

The particle size distribution of metals NPs, as showed in Fig. 9, was analyzed using a particle size analyzer. The average size distribution of FeNPs (Fig. 9A), CuNPs (Fig. 9B), CoNPs (Fig. 9C), and ZnNPs (Fig. 9D) were in range 1–3 nm, 8–18 nm, 8–22, and 4–16 nm respectively.

#### Energy-dispersive X-ray spectroscopy (EDX)

EDX analysis gives qualitative as well as quantitative status of elements that may be involved in the formation of NPs and proves the ability of bacterial cells to reduce and accumulate metals from the culture medium. The high presence of Fe, Cu, Co, and Zn in analyzed bacterial cells, as observed in EDX analysis, is reported in Table 3. Iron contents in *P. putida* isolate was 33%, the content of copper in *E. thailandicus* isolate was 39.2%, the content of cobalt in *M. hydrocarbonoclasticus* was 32% and zinc in *P. geniculata* was 39.1%. The presence of P, Cd, Al, K, Si, Na, Mg, Cl, and S as debris of bacterial cells was observed.

**Table 3:** Energy-dispersive X-ray analysis of *Enterococcus thailandicus* E12 cultured on copper nitrate, *Marinobacter hydrocarbonoclasticus* M 69 cultured on cobalt nitrate, *Pseudomonas putida* T65 cultured on iron nitrate and *P. geniculata* G 80 cultured on zinc nitrate.

Element%	Fe Iron	Cu Copper	Co Cobalt	Zn Zinc	P Phosphorus	Ca Calcium	Al Aluminum	K Potassium	Si Silicon	Na Sodium	Mg Magnesium	Cl Chlorine	S Sulfur
Isolate													
*E. thailandicus*	–	39.2	–	3.1	22.6	10.2	1.0	1.7	0.7	8.8	–	0.3	7.6
*M. hydrocarbonoclasticus*	10.5	–	32	2.5	19.21	–	3.12	7.5	–	–	–	16.6	1.25
*P. putida*	33	–	–	14.36	12.56	6.1	1.5	3.4	–	15.2	0.6	–	7.2
*P. geniculata*	1.45	1.1	–	39.1	11.7	12.2	–	5.2	0.2	11.7	1.2	0.6	9.4

#### Vibrating sample magnetometer (VSM) analysis

**Figure 10:**
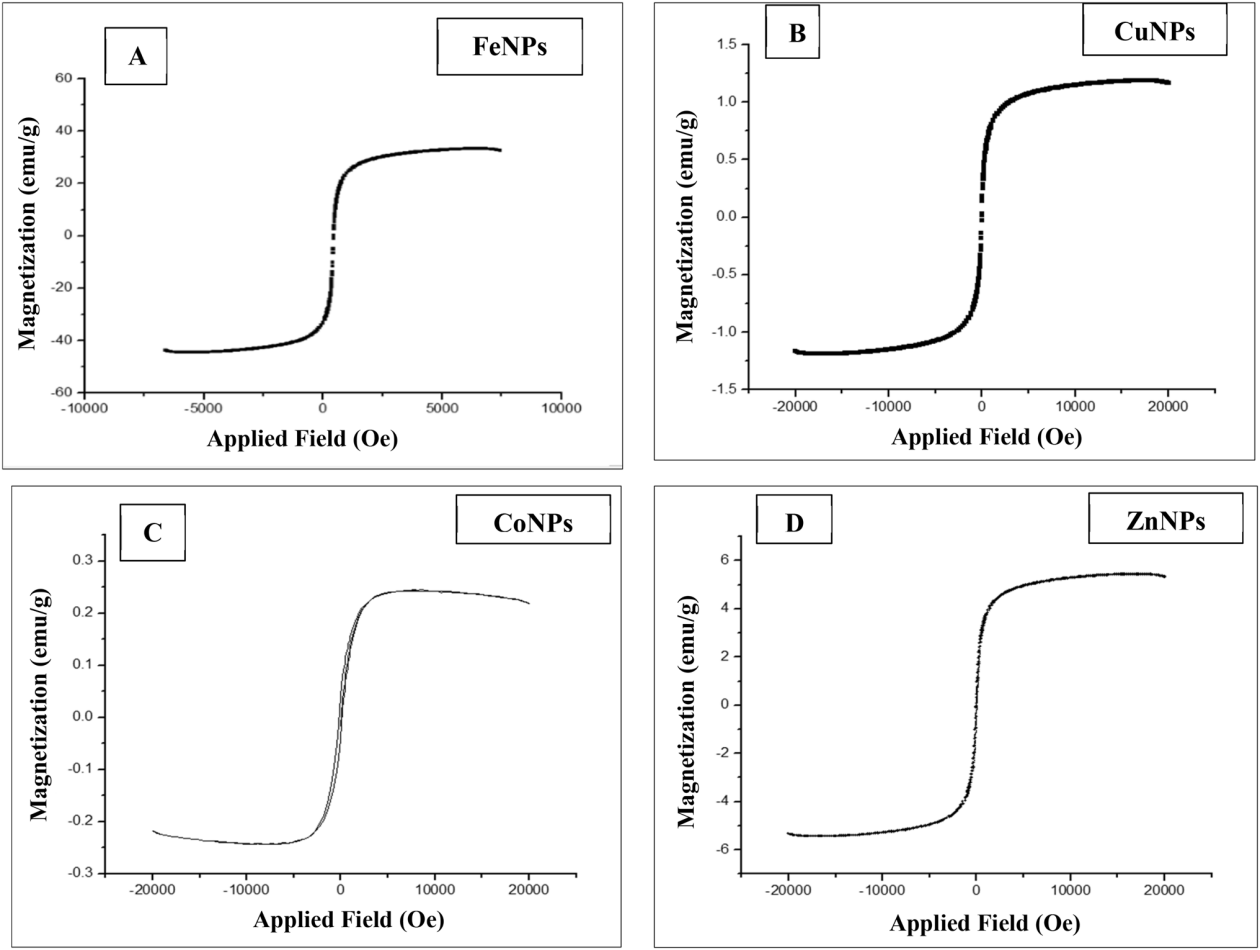
Room temperature magnetization curves of FeNPs (**A**), CuNPs (**B**), CoNPs (**C**) and ZnNPs (**D**).

Magnetic characterization of metallic NPs was performed using a **VSM**. Plots of magnetization versus the magnetic field at 300 K for Fe, Cu, Co, and ZnNPs are shown in Fig. 10.

As shown in Fig. 10A the plots of the FeNPs measured a saturation magnetization value around 36.9 emu/g with no hysteresis, the magnetic characterization of CuNPs, as shown in Fig. 10B indicated, low magnetic behavior with saturation magnetization value around 1.3 emu/g without any hysteresis. Magnetization curve of CoNPs showed a very low value around 0.2–0.3 emu/g (Fig. 10C), and ZnNPS saturation magnetization value was` around 5 emu/g as shown in Fig. 10D.

#### Inductively coupled plasma-optical emission spectroscopic analysis (ICP-OES)

The bacterial bio-sorption and reduction of metals capability was assayed by ICP-OES. Determination of Residual Metal was measured in cell-free supernatant compared with the control medium supplemented with metals. The ability of the selected bacterial isolates to accumulate metals intracellularly is shown in S (4). Bacterial isolate *P. geniculata* can accumulate all amount of Zn^2+^ within 2 days (S4D), followed by *P. putida* that can accumulate all amount of Fe^3+^ from the culture medium within 4 days (S4A), while isolates *E. thailandicus* and *M. hydrocarbonoclasticus* can accumulate Cu^2+^ (S4B) and Co^2+^ (S4C) within 5 and 6 days sequentially.

### Effect of metals NPs on some phytopathogenic bacteria in vitro

#### Determination of inhibition zone (IZ)

The antibacterial effect of metallic NPs was determined by the well diffusion method. The mean value of IZ is reported in Table 4, while an example image is shown in S5. FeNPs revealed the highest significant means of IZ 35.79, 34.02, and 33.44 mm against *Pectobacterium carotovorum* subsp. *carotovorum, Agrobacterium tumefaciens,* and *Dickeya solani* respectively, while *Ralstonia solanacearum* showed the smallest means of IZ 26.55 mm. FeNPs at the concentration of 300 µg/mL had the highest mean value of IZ (42.93 mm) followed by 200 µg/mL (39.05 mm) comparing with other tested concentrations.

**Table 4:** Antibacterial activity of Fe, Cu, Co and Zn Nanoparticles (NPs) on the growth of some phytopathogenic bacteria.

NPs	Concentration	1000 µg/mL	700 µg/mL	500 µg/mL	300 µg/mL	200 µg/mL	100 µg/mL	Means
	Isolates							
Iron (Fe)	*P. carotovorum* subsp. *carotovorum*	*12.78^w^	21.38^r^	49.39^a^	46.29^b^	45.10^c^	39.83^g^	35.79a
	*D. solani*	11.89^xy^	16.63^v^	44.76^cd^	46.77^b^	44.23^d^	36.41^j^	33.44c
	*E. cloacae*	11.41^y^	12.27^wx^	39.19^h^	44.60^cd^	41.20f.	41.31f.	31.66d
	*P. atrosepticum*	16.32^v^	17.40^u^	25.33^p^	40.42^g^	38.47^i^	30.35^m^	28.04e
	*R. solanacearum*	12.27^wx^	12.53^w^	33.30^k^	35.86^j^	31.94^l^	33.42^k^	26.55f.
	*E. amylovora*	18.37^t^	19.37^s^	26.83^o^	42.03^e^	32.14^l^	29.34^n^	28.01e
	*A. tumefaciens*	22.43^q^	26.34^o^	32.32^l^	44.57^cd^	40.27^g^	38.20^i^	34.02b
Means		15.06^c^	17.98^c^	35.87^b^	42.93^a^	39.05^ab^	35.55^b^	
Copper (Cu)	*P. carotovorum* subsp. *carotovorum*	21.31^u^	31.95^lm^	45.57^a^	42.32^b^	40.39^d^	36.77^g^	36.38a
	*D. solani*	22.36^t^	41.19^c^	40.24^d^	39.47^e^	35.06^i^	26.50^q^	34.13b
	*E. cloacae*	17.28^w^	32.27^l^	36.12^h^	34.41^j^	30.20^no^	19.46^v^	28.29e
	*P. atrosepticum*	11.23^z^	25.22^r^	35.20^i^	34.13^j^	32.14^l^	22.24^t^	26.69f.
	*R. solanacearum*	16.54^x^	23.42^s^	38.32f.	38.23f.	34.23^j^	31.42^m^	30.36d
	*E. amylovora*	22.46^t^	30.77^n^	42.28^b^	40.34^d^	38.97^e^	21.35^u^	32.69c
	*A. tumefaciens*	15.11^y^	28.38^p^	33.18^k^	29.83^o^	25.52^r^	15.49^y^	24.58 g
Means		18.04^d^	30.45^b^	38.70^a^	36.96^a^	33.78a^b^	24.74^c^	
Cobalt (Co)	*P. carotovorum* subsp. *carotovorum*	*10.53^w^	25.34^mn^	39.49^a^	33.30^d^	27.62^j^	18.25^t^	25.75b
	*D. solani*	21.78^pq^	24.33^o^	37.28^b^	34.75^c^	22.25^p^	20.21^r^	26.76a
	*E. cloacae*	14.01^v^	22.36^p^	31.67^fg^	28.39^i^	25.09^n^	16.22^u^	22.95d
	*P. atrosepticum*	11.06^w^	21.50^q^	32.44^e^	25.25^n^	26.95^jk^	13.88 ^v^	21.84e
	*R. solanacearum*	15.53^u^	26.36^kl^	33.55^d^	30.60^h^	24.17^o^	14.16 ^v^	24.06c
	*E. amylovora*	14.30^v^	24.30^o^	31.05^gh^	31.78^ef^	19.48^s^	11.12^w^	22.00d
	*A .tumefaciens*	9.14^w^	22.20^pq^	29.06^i^	27.29^j^	25.97^lm^	19.28^s^	22.15d
Means		13.76^c^	23.77^b^	33.50^a^	30.19^a^	24.50^b^	16.16^c^	
Zinc (Zn)	*P. carotovorum* subsp. *carotovorum*	26.28^b^	25.0^c^	21.16f.	16.18^j^	12.49^n^	0.0^q^	16.85b
	*D. solani*	27.84^a^	28.41^a^	22.39^e^	19.18^g^	14.49^l^	0.0^q^	18.71a
	*E. cloacae*	21.22f.	16.12^j^	14.34^l^	14.25^l^	10.55^p^	0.0^q^	12.74c
	*P. atrosepticum*	18.99^g^	13.62^m^	10.69^p^	10.32^p^	0.0^q^	0.0^q^	8.936c
	*R. solanacearum*	24.28^d^	21.90^e^	17.31^i^	15.13^k^	15.30^k^	0.0^q^	15.65b
	*E. amylovora*	26.04^b^	21.97^e^	19.51^g^	18.21^h^	15.30^k^	0.0^q^	16.83b
	*A. tumefaciens*	19.23^g^	13.91^lm^	11.29^o^	11.45^o^	0.0^q^	0.0^q^	9.313c
Means		23.41^a^	20.13^ab^	16.67^bc^	14.96^c^	9.73^d^	0^e^	

Statistically significant at p ≤ 0.05, Means with Common letters are not significant (i.e. Means with Different letters are significant), * Inhibition zone (IZ) diameter (mm).

IZ mean values recorded for CuNPs against *P. carotovorum* subsp. *carotovorum, D. solani* and *Erwinia amylovora* were 36. 38, 34.13, and 32.69 mm sequentially, followed by *R. solanacearum* (30.36 mm), while the smallest mean of IZ (24.58 mm) was against *A. tumefaciens*. CuNPS concentration of 500 µg/mL gave the highest mean of IZ (38.70 mm) followed by 300 µg/mL (36.96 mm).

CoNPs and ZnNPs exhibited a lower antibacterial effect than FeNPs and CuNPs. CoNPs showed the highest IZ mean, 26.76 mm, against *D. solani*, and the smallest means of IZ (22.00 mm, 21.84 mm respectively) against *E. amylovora* and *Pectobacterium atrosepticum*. CoNPs at the concentration 500 µg/mL gave the highest mean of IZ (33.50 mm) followed by 300 µg/mL (30.19 mm).

ZnNPs versus *D. solani* showed the highest mean of IZ (18.71 mm), while the lowest mean of IZ (16.85 mm) was the one against *P. atrosepticum*. ZnNPs at the concentration of 1000 µg/mL gave the highest mean of IZ (23.41 mm), followed by the concentration of 700 µg/mL (20.13 mm).

#### Determination of MIC and MBC of metals NPs

To investigate the growth inhibition effect of NPs quantitatively against various plant bacterial pathogens, the MIC and MBC were measured and results are reported in Table 5. It was demonstrated that FeNPs, CuNPs, and CoNPs could completely inhibit the growth of tested isolates at a low concentration (50 and 100 µg/mL), while ZnNPs proved to inhibit the growth of *P. carotovorum* subsp. *carotovorum, E. colacae, R. solanacearum, E. amylovora* at 200 µg/mL and the growth of *P. atrosepticum,* and *A. tumefaciens* at 400 µg/mL. MBC values of 50, 100, 200 µg/mL were recorded for FeNPs, CuNPs, CoNPs against *D. solani, P. carotovorum* subsp. *carotovorum,* and *R. solanacearum.* ZnNPs MBC values were 200 µg/mL against *P. carotovorum* subsp. *carotovorum, D. solani*, and *E. cloacae*, 400 µg/mL against *R. solanacearum* and *E. amylovora* and 800 µg/mL against *P. atrosepticum* and *A. tumefaciens*.

**Table 5:** Minimum inhibitory concentrations (MICs) and minimum bactericidal concentrations (MBCs) of tested metals NPs against some phytopathogenic bacteria.

Concentration	FeNPs		CuNPs		CoNPs		ZnNPs	
Isolates	MIC (µg/mL)	MBC (µg/mL)	MIC (µg/mL)	MBC (µg/mL)	MIC (µg/mL)	MBC (µg/mL)	MIC (µg/mL)	MBC (µg/mL)
*P. carotovorum* subsp*. carotovorum*	50	100	100	100	50	100	200	200
*D. solani*	50	50	50	50	50	100	200	200
*E. cloacae*	50	100	50	100	100	200	200	200
*P. atrosepticum*	100	100	100	100	100	200	400	800
*R. solanacearum*	100	200	100	100	100	100	200	400
*E. amylovora*	50	100	100		50	100	200	400
*A .tumefaciens*	100	200	100	100	100	200	400	800

## Discussion

Water samples were collected from different locations in Egypt, industrial wastewater, seawater, wastewater, and lake water. The chemical analysis demonstrated that samples contained inorganic pollutants such as Fe^3+^, Zn^2+^, Cu^2+^, and highly toxic heavy metals like Cd^2+^ and Pb^2+^. Nano-metals forming bacteria that could be adapted to detoxify these heavy metals were isolated from collected samples. Different studies reported that microorganisms have developed the capabilities to protect themselves from heavy metal toxicity by various mechanisms such as adsorption, uptake, methylation, oxidation, and reduction^20^.

Metal-reducing bacteria were isolated with dark brown colony or dark zone around bacterial growth. Lima de Silva et al*.*^20^ reported that the changes observed in the colony colors were due to chemical modification of metals when interacting with the bacteria, and not to the induction of real pigmentation. Zaki et al.^21^ reported that color change in *Stenotrophomonas rhizophila* grown at silver nitrate indicates to reduction reaction of AgNo_3_ and formation of AgNPs. The present study concluded that all selected bacterial isolates could be able to produce nitrate reductase enzyme. The nitrate reductase was reported to be responsible for nanoparticles (NPs) production especially AgNPs^22,23^.

The morphological, biochemical, and physiological behavior of 4 tested isolates confirmed that two isolates belonged to *Pseudomonas* spp. and the other two isolates belonged to *Enterococcus* sp. and *Marinobacter* sp*.* Molecular approaches, i.e. 16S rRNA sequencing and phylogenetic analysis of selected isolates, revealed similarity to *P. putida*, *E. thailandicus*, *M. hydrocarbonoclasticus*, and *P. geniculata*. The literature reported the use of *P. putida* for bioremediation, due to its ability to degrade organic solvents such as toluene^24^, and for silver and selenium NPs synthesis^25^. Tang et al*.*^26^ reported that *P. geniculata* was capable of efficiently degrading nicotine. *E. thailandicus* was first isolated from fermented sausage in Thailand in 2008 and can produce L-lactic acid^27^. On the other hand, *M. hydrocarbonoclasticus* which use nitrate (NO^3−^) or nitrite (NO^2−^) as the terminal electron acceptor to form gas product such as N_2_O, was first isolated near a petroleum refinery in the Mediterranean Sea.

UV–Vis spectroscopy used for characterization of metal NPs, had proven to be a very useful technique for monitoring the signature of colloidal particles, especially for noble metal since they exhibit strong surface plasmon resonance absorption in the visible region and are highly sensitive to the surface modification^28^. Present data showed the absorption maximum peak of FeNPs, CoNPs, CuNPs, and ZnNPs in agreement with the results of Zhang and Lan^29^, Yuvakkumar et al.^30^, Annapurna et al.^31^ and Devasenan et al.^32^.

All UV absorption peaks in the range from 200 to 600 nm, characterized by the absence of long tailing on large wavelengths suggested the absence of aggregation between particles^33^. The presence of only a single band at such wavelength ranges reflected the presence of small spherical particles according to Mie's theory^34^. The position and shape of the surface plasmon absorption of noble metal nanoclusters were strongly dependent on the particle size, dielectric medium and surface adsorbed species^35^.

Data obtained from TEM concluded that the selected isolates were able to detoxify and reduce metallic ions to nanoscale particles. FeNPs in the cytoplasm and periplasmic space of *P. putida* was observed. Such results were in agreement with Varshney et al.^36^**,** who found that *P. stutzeri* synthesized silver NPs within its periplasmic space. Synthesis of CuNPs was shown in the cytoplasm space of *E. thailandicus*, also particles were aggregated in the center of cells by the action of proteins of cytoplasm which prevent their release to the surrounding media through the cell wall. CoNPs synthesis in the case of isolate *M. hydrocarbonoclasticus *appeared diffused in the cytoplasm. ZnNPs appeared in periplasmic and cytoplasmic space of *P. geniculata*. Iravani^37^ explained that the ability of bacteria to survive and grow in stressful situations might be due to specific mechanisms of resistance which include: efflux pumps, metal efflux systems, inactivation of metals, impermeability to metals, the lack of specific metal transport systems, alteration of solubility, toxicity by changes in the redox state of the metal ions, intracellular precipitation of metals, and volatilization of toxic metals by enzymatic reactions. On the other hand, Gomathy and Sabarinathan^38^ suggested that cellular mechanisms may be implicated in the resistance and tolerance of microorganisms to excessive concentrations of heavy metals in the environment. The strategy adopted include slow transport into the cell, detoxification or incorporation of specific metals into enzymes.

Deepak et al*.*^39^ reported the process that involves the reduction of metals ions. The first step involves the interaction between these metal ions with nitrate reductase present inside the cell (periplasmic and cytoplasmic membrane) and their bioreduction to metallic form. The accumulation of intracellular NPs was reported in different studies like the synthesis of silver NPs by *Stenotrophomonas rhizophilia*^21^, *E. coli,* palladium NPs by *Desulfovibrio desulfuricans and Bacillus benzeovorans*^40^**,** and AuNPs by *Lactobacillus kimchicus*^41^**.**

Results of characterization of size and morphology of metals NPs*,* based on TEM agreed with previous studies for FeNPs in the range of 1–4 nm^42^, CuNPs in the range 8–14 nm^43^, CoNPs in the range 9–20 nm and ZnNPs in the range 4–13 nm^44^. Also, in agreement with previous studies the PSA analysis showed that the range size distribution of FeNPs was 1–3 nm^45^, CuNPs in range 8–18 nm^46^, CoNPs in range 8–22 nm^47^ and ZnNPs in range 4–16 nm^48^.

Patterns of XRD were analyzed to determine peak intensity, position, and width. The diffraction peaks of all NPs appeared sharp, and clearly distinguishable, which indicates the ultra-fine nature and small crystallite size. The XRD spectrum contained no other phase, indicating the purity of the sample^49^. The XRD patterns correspond to FeNPs diffractogram, in which the characteristic peak of highest intensity occurred at 45.50° correspond to (111), crystallographic planes of face-centered cubic iron zerovalent crystals^50^. Two main characteristic diffraction peaks for CuNPs were observed at around 2θ = 42°, 50° which correspond to the (111), (200) crystallographic planes of face-centered cubic Cu^2+^ phase (JCPDS No.04-0784)^51^. The XRD patterns in of CoNPs show the diffraction peaks at around 43.2°, 54.5°, and 77.9° could be assigned to the (111), (200), and (220) planes of the cubic Co nanocrystal^52^**.** ZnNPs showed that all the diffraction peaks could be well indexed to the characteristics of the cubic of ZnNPs. The sharp and narrow diffraction peaks appeared at about 2θ of 43, 56 and 74.83°, and were assigned to (111), (200), and (220) plane values of ZnNPs. This diffraction pattern has corresponded to pure Zn Nanopowder. A small peak is also observed at around 36° indicated that a small amount of zinc was oxidized and converted into zinc oxide^32^**.** The XRD pattern of all metallic NPs indicated that the metals NPs had a spherical structure with a small size. The obtained results illustrated that metal ions had indeed been reduced to Nano-form by reducing enzyme of bacterial isolates and prof the ability of selected bacterial isolates to produce metals NPs^9^.

Raman spectroscopy is especially useful if minerals are to be identified that are poorly defined or that cannot easily be distinguished using other methods such as XRD. In this study, Raman spectra of metal NPs showed that the 3 Raman spectra of FeNPs had mainly 3 bands observed around 200, 290, and 1100 cm^−1^. Raman region of interest in this investigation was around 200–1100 cm^-1^, and it was confirmed to Fe zerovalent Fe^0^ NPs peaks. These results were matched with Dong et al.^50^. Besides, results of Raman spectra of CuNPs with peak absorptions around 500 cm^−1^, 1250 cm^−1^, and 1500 cm^−1^ indicated that the sample contains Cu^0^ NPs^53^. Raman spectra showed absorptions peak around 500 cm^−1^ and 1000 cm^−1^ and it corresponded with the Raman shift pattern of the CoNPs^52^. In addition to Raman spectra with 2 strong peaks around 300–500 cm^−1^ and 1400 cm^−1^, these peaks characteristics were identical to peaks of ZnNPs^54^. All Raman spectra of samples indicated the high quality of metal NPs, as well as, these results were compatible with XRD analyses.

Analysis using EDX indicated that the content of Fe in *P. putida* isolate was 46%**,** the content of Cu in *E. thailandicus* was 47%, which was coherent with the results by Eltarahony et al.^55^. Content of Co in *M. hydrocarbonoclasticus* was 41%, and Zn in *P. geniculata* was 59%, in agreement with finding by Eltarahony et al.^56^. These results fixed that all bacterial isolates could accumulate and reduce the high amount of metal for NPs intracellular synthesis. The presence of other metals might be referred to some elements in bacterial cell structure and biomolecules such as DNA, ATP, RNA, and amino acids^19^.

The magnetic properties of the metal NPs have been investigated using a VSM. The plots of the FeNPs indicate a superparamagnetic behavior at room temperature which is compatible with Eivari et al.^57^, with no hysteresis. The measured saturation magnetization value around 36.9 emu/g. The magnetic characterization of CuNPs indicated low magnetic behavior with a saturation magnetization value of around 1.3 emu/g without any hysteresis^58^**.** Also, magnetization curves of CoNPs were very low around 0.2–0.3 emu/g, with a soft hysteresis loop^59^. In addition to the magnetization curve of ZnNPs with saturation magnetization had a value of around 5 emu/g^60^. From these results, we concluded that Fe, Cu, and Zn were paramagnetic NPs, and Co was ferro-magnetic NPs.

Determination of residual metal concentration using ICP-OES demonstrated that selected bacterial isolates could detoxificate and accumulates metals intracellular. *P. putida* could accumulate all amount of Fe^3+^ from the culture medium within 4 days, as well *P. geniculata* could accumulate all amount of Zn^2+^ within 2 days, and it might be referring to the ability degree of isolates to detoxification of metals and the toxicity level of metals like Cu^2+^ and Co^2+^. Different studies reported the importance of Fe^3+^ and Zn^2+^ to bacterial cells and their role in different biological pathways. Ahmad et al.^61^ reported that Zn^2+^ plays a significant role in cellular division and protein synthesis contributes to carbohydrate, lipid, and nucleic acid metabolism**.** Fe^3+^ participates in a large number of cellular processes, the most important of which were oxygen transport, ATP generation, cell growth, proliferation, and detoxification. It was a coenzyme or enzyme activator of ribonucleotide reductase, a key enzyme for DNA synthesis, which catalyzes the conversion of ribonucleotides to deoxyribonucleotides and particularly of deoxyuridine to thymidine. ICP-OES results were compatible and confirm ability of selected isolates to reduce and accumulate metals^19^**.**

The antibacterial effect of metal NPs against some phytopathogenic bacteria noted that in case of low concentrations of FeNPs**,** CuNPs and CoNPs increase the antibacterial effect and this effect significantly decreased with high concentrations. Rout et al.^62^ declared that size of the inhibition zone increased significantly with decreasing the size of the NPs. It is reasonable to state that binding of the NPs to the bacteria depends on the surface available for interaction. They added that smaller and monodispersed particles having the larger surface area available for interaction would give more of a biocidal effect than the aggregated and larger particles**.** Moreover, present data revealed that the antibacterial potential increased with an increase in the concentration of ZnNPs.

Several studies reported the antibacterial effect of metals NPs and their antibacterial mechanisms. Lee et al.^63^ reported the inactivation of *E. coli* by zero-valent Fe, furthermore, NPs leads to the production of reactive oxygen species (ROS), resulting in the generation of hydroxyl radicals (OH^−^) from superoxide (O^2−^) and hydrogen peroxide (H_2_O_2_) in microbial cells. These radicals promote oxidative stress and cause cell membrane damage, which contributes to the outflow of intracellular contents and, finally, cell death due to the penetration of the small NPs (sizes ranging from 10–80 nm) into *E. coli* membranes^64^. Other NPs such as ZnO-NPs^65^, Aerogel-MgO-NPs^66^, and TiO2-NPs^67^ have been reported also to cause a loss of membrane integrity and leakage. Other studies on ZnO-NPs and MgO-NPs concluded that antibacterial activity increased with decreasing particle size^68^. Moreover, researches had demonstrated that the small size of NPs, which have characteristic dimensions < 100 nm, can contribute to the bactericidal effect. Their uniquely small size results in novel properties, like the greatest interaction with cells due to a larger surface area-to-mass ratio and multilateral and controllable application^69^. The antibacterial properties of CuO NPs have been investigated, which were found that able to cause protein oxidation, lipid peroxidation and DNA degradation in *E. coli* cells^70^. Khalil et al.^71^ reported that synthesized cobalt oxide NPs were studied for their antibacterial potential against 3 G^**-**^ve (*Pseudomonas aeruginosa, Klebsiella pneumonia,* and *Escherichia coli*) and 3 G^**+**^ve bacterial strains (*Staphylococcus epidermis*, *Staphylococcus aureus* and *Bacillus subtilis*). It was noted that the antibacterial potential increased with an increase in the concentration of the nanoparticles.

In this study, FeNPs**,** CuNPs, and CoNPs were found to be effective at 50–100 µg/mL against some phytopathogenic bacteria, likewise, 200–400 µg/mL of ZnNPs were effective. This effectiveness at lower concentrations might be referred to the small size of bacterial synthesized metal NPs. It was reported that ultrafine particle size causes its action at a lower concentration, whereas our study used NPs with a size range of 2–10 nm. A previous study by Niemirowicz et al.^72^ indicated that MIC and MBC of FeNPs against *Staphylococcus aureus* and *P. aeruginosa* was 64, 182 µg/mL and 128, 265 µg/mL on the relay, also Ruparelia et al.^73^ noted that MIC and MBC of CuNPs against *E. coli* was 140, 160 µg/mL. On the other hand, Aysa and Salman^74^ demonstrated that 3.7 µg/mL was the MIC and MBC value of ZnNPs against *P. aeruginosa.*

## Conclusion and recommendations:

From this study, we concluded that biological synthesis of NPs using bacterial cells is eco-friendly, fast, and inexpensive. The toxicity of FeNPs, CuNPs, CoNPs, and ZnNPs to phytopathogenic bacteria in addition to their bactericidal effect, recommend future investigations on the toxicity and safety concentrations of metal NPs to animals and human cells. These future studies seem to be necessary to be performed, in order to precise for toxicity prevention and the applicability of metals NPs in bactericide industry.

## Material and methods

### Samples collection

Various environmental water samples showing harsh conditions for microbial growth (industrial wastewater, seawater, wastewater, and lake water), containing organic and inorganic pollutants were collected from several diverse habitats (Alexandria, Hurghada, and Damietta Governorates), during April 2015 (Table 6).

**Table 6:** Sample code, location (Governorate) and sample type used for isolation of metal NPs forming bacteria.

Sample code	Location (governorate)	Sample type
A1	Petroleum company (Damietta)	Industrial waste water
A2	Port of Eastern (Alexandria)	Sea water
A3	Port of Ras sedr (Red Sea)	Sea water
A4	East of the Gulf of Suez (red sea)	Sea water
A5	Aboukir Bay (Alexandria)	Sea water
A6	Tolombat -Almax (Alexandria)	Waste water
A7	Mariout Lake (Alexandria)	Lake water
A8	Port of Jebel Al Azeet (Red Sea)	Sea water

### Physicochemical analysis of water samples

pH, NO_2_^−^, NO_3_^−^, salinity, Fe^3+^, Zn, Cu^2+^, Pb^2+^, and Cd^2+^ were analyzed and performed according to APHA^75^.

### Isolation of Nano-metals forming bacteria

Each water sample (1 mL) was inoculated in nutrient broth (NB), and then taken in a series of 250 mL Erlenmeyer flasks containing 49 mL of NB. In the case of seawater samples, 1 mL of each sample was inoculated in Marine Broth (2216)^76^ and incubated at 30 °C for 24 h at 150 rpm in an orbital shaker. Samples were serially diluted in 0.8% saline then plated on Luria Bertani (LB) agar plates supplemented with 5 mM of either Fe (NO_3_)_3_·9H_2_O, Cu (NO_3_)_2_. 3H_2_O, Co (NO_3_)_2_·6H_2_O and Zn (NO_3_)_2_·6H_2_O (Sigma Aldrich) and incubated at 30 °C for 6 days. Plates were examined daily for dark colonies and/or with a dark zone on the agar. The obtained pure bacterial colonies, through a single colony isolation technique^77^, were maintained by mixing bacterial suspension in 20% glycerol and stored at − 80 °C for further studies**.**

### Determination of nitrate reductase

For crude enzyme determination**,** isolates were grown on LB broth supplemented with 3.5 mM of Fe^3+^, Cu^2+^, Co^2+^, and Zn^2+^ nitrate separately, then cells were collected and disrupted by an Ultrasonic generator (Vibra-Cell Ultrasonic Liquid Processors Sonics and Materials VC 505 / VC 750160 W) for 5 min. Nitrate reductase activity was assayed according to Antonio et al.^78^.

### Morphological, physiological, and biochemical identification tests

Motility, Gram stain, spore formation were done for Morphological tests. Physiological tests for the effect of different pH values on growth, temperature, and NaCl tolerance as well as gelatin liquefaction was determined according to Bergey's Manual of Determinative Bacteriology^79^**.** For testing the ability of isolates to grow under anaerobic conditions, bacterial isolates were tested using AnaeroGen jar 2.5 liters^80^**.**

Biochemical characterization tests were performed using diagnostics ENC 8 kit and GN 24 kit identification system for Gram-positive and Gram-negative bacteria, respectively. As well DNAase test was performed by streaking each bacterial culture on the DNAase agar plate (HIMEDIA M482) and incubated at 28 ± 2 °C for 3 days, then the plate was flooded with 0.1% toluidine blue. A clearance zone around the colonies was recorded as a positive reaction^81^.

### Molecular identification of NPs forming bacteria through 16S rRNA gene

For DNA extraction protocol from bacterial cells, an aliquot of 1 mL of AMSHAGE DNA extraction kit was added and followed the steps of Abd-El-Haleem^82^. PCR amplification of the 16S rRNA gene was performed for 4 isolates on a "Biometra PCR Thermocycler" using one pair of primer 16S-F (27F) AGAGTTTGATCMTGGCTCAG and 16S-R(1492R) GATTACCTTGTTACGACTT according to Wang et al.^83^.

### PCR products electrophoresis and visualization

Ten μL of PCR product was loaded per gel slot. Electrophoresis was performed at 100 Volt with (0.5 x) Tris–Acetate—EDTA buffer (TAE) [Tris base, 108 g/L; acetic acid, 55 g/L and 0.5 M EDTA with a pH of 8] as running buffer in 1.5% agarose gel cast in 0.5 × (TAE buffer). The gel was stained in (1 μL) ethidium bromide (Royal Biogene). Finally, the gel contained PCR product was visualized with a syngeneic gel documentation system.

### Purification of PCR product and sequencing of 16S rRNA gene

PCR products were purified using NEPRAS DNA purification kit^84^. Partial DNA sequencing was performed for the PCR amplified 16S rRNA gene using an ABI PRISM dye terminator cycle sequencing kit with Ampli Taq DNA polymerase and an Applied Biosystems 373 DNA Sequencer (Perkin-Elmer, Foster City, Calif.).

### Alignment and phylogenetic analysis

Bootstrap neighbor-joining tree was generated using MEGA version 6.1 from CLUSTALW alignment. Comparisons with sequences in the GenBank database were achieved in BLASTN searches at the National Center for Biotechnology Information (NCBI) site (http://www.ncbi.nlm.nih.gov).^85^ Production and Extraction of metals nanoparticles (NPs) were performed according to Zaki et al.^86^ and Kamal et al.^87^ respectively.

### Characterization of metals NPs

Characterization was performed using a variety of analytical techniques. Ultraviolet–Visible spectroscopy (UV–Vis), X-ray diffraction (XRD)^88^, Transmission electron microscope (TEM)^89^, Raman Spectroscopy^90^, Particle size distribution (PSA)^45^, Energy dispersive X-ray (EDX) spectroscopy^85,91^, Vibrating Sample Magnetometer (VSM)^17^ and Inductively Coupled Plasma-Optical Emission Spectroscopy (ICP-OES)^92^ were carried out for characterization of metals NPs.

### Application of metals NPs on some phytopathogenic bacteria in vitro

The metals NPs were evaluated for antibacterial activity against 7 molecular identified phytopathogenic bacteria (Table 7). Determination of the inhibition zone (IZ) of tested phytopathogenic bacteria was done by a well diffusion method^93^. The minimum inhibitory concentration (MIC) was determined based on batch cultures containing varying concentrations of metals NPs with serial two-fold dilution in suspension (25–800 µg/mL). All the experiments were carried out in triplicate. Also, the determination of the minimum bactericidal concentration (MBC) was performed as suggested by Avadi et al*.*^94^. To test for bactericidal effect, one mL of each culture flask which was used in the MIC experiment was plated in duplicates on LB agar free of NPs and incubated at 30 °C for 48 h. NPs concentration causing the bactericidal effect was selected based on the absence of colonies on the agar plate^95^.

**Table 7:** Phytopathogenic bacterial isolates used in this study.

Bacterial isolates	Accession No
*Pectobacterium carotovorum* subsp. *carotovorum*	Acc. No. LN811442^96^
*Dickeya solani*	Acc. No. LT592259^96^
*Enterobacter cloacae*	Acc. No. LT592256^96^
*Pectobacterium atrosepticum*	Identified by specific primer (Y45,Y46), Bacterial Pl. Dis. and Molecular Bacteriology lab., Dept. of Pl. Pathology, Fac. of Agric., Alex. Uni. Egypt
*Ralstonia solanacearum*	Acc. No. LN681200^97^
*Erwinia amylovora*	Acc. No. LN875713^98^
*Agrobacterium tumefaciens*	Acc. No. LT630451^99^

## Supplementary Information

Supplementary Information.

## Data Availability

The datasets used as well as the materials are available in this study.

## References

[1] Islam W, Noman A, Qasim M, Wang L (2018). Plant responses to pathogen attack: Small RNAs in focus. Int. J. Mol. Sci..

[2] Shoeib AA, Hassanein FM (1994). Detection of epiphytic populations of streptomycin sensitive and resistant strains of *Erwinia amylovora* and control of fire blight disease in Egypt. Alex. J. Agric. Res..

[3] Buttimer C, McAuliffe O, Ross RP, Hill C, O’Mahony J, Coffey A (2017). Bacteriophages and bacterial plant diseases. Front. Microbiol..

[4] Monaco JT, Weller SC, Ashton FM (2002). Herbicide registration and environmental impact Weed science, principles and practices.

[5] Ocsoy I, Paret ML, Ocsoy MA, Kunwar S, Chen T, You M, Tan W (2013). Nanotechnology in plant disease management*,* DNA-directed silver nanoparticles on graphene oxide as an antibacterial against *Xanthomonas perforans*. Am. Chem. Soc. Nano.

[6] Alsayed MFS, Shoeib AA, Hindi AA, Awad MA, Ortashi KDMO (2015). Synthesis of silver nanoparticles discourage the growth of isolated bacteria invading the blood stream. Dig. J. Nanomater. Biostruct..

[7] Reverberi AP, Salerno M, Lauciello S, Fabiano B (2016). Synthesis of copper nanoparticles in ethylene glycol by chemical reduction with vanadium (^+2^) salts. Materials (Basel).

[8] Irshad R, Tahir K, Li B, Ahmad A, Siddiqui AR, Nazir S (2017). Antibacterial activity of biochemically capped iron oxide nanoparticles: A view towards green chemistry. J. Photochem. Photobiol. B.

[9] Subhan A, Irshad R, Nazir S, Tahir K, Ahmad A, Khan AU, Khan ZUH (2020). A new study of biomediated Pd/tiO_2_: a competitive system for *Escherichia coli* inhibition and radical stabilization. Mater. Res. Exp..

[10] Kulkarni N, Muddapur U (2014). Biosynthesis of metal nanoparticles: A review. J. Nanotechnol..

[11] Lin PC, Lin S, Wang PC, Sridhar R (2014). Techniques for physicochemical characterization of nanomaterials. Biotechnol. Adv..

[12] Sapsford KE, Tyner KM, Dair BJ, Deschamps JR, Medintz IL (2011). Analyzing nanomaterial bioconjugates*,* a review of current and emerging purification and characterization techniques. Anal. Chem..

[13] Popovic Z, Dohčević-Mitrović Z, Scepanovic M, Grujić-Brojčin M, Aškrabić S (2011). Raman scattering on nanomaterials and nanostructures. Ann. Phys..

[14] Kattumenu R, Lee C, Bliznyuk V, Singamaneni S, Kumar CSR (2012). Micro-Raman spectroscopy of nanostructures. Raman Spectroscopy for Nanomaterials Characterization.

[15] Bernier MC, Besse M, Vayssade M, Morandat S, El Kirat K (2012). Titaniumdioxide nanoparticles disturb the fibronectin-mediated adhesion and spreading of pre-osteoblastic cells. Langmuir.

[16] Williams, D.B. & Carter, C.B. (2009). The Transmission electron microscopy. In: Williams, D.B. & Carter, C.B. Transmission Electron Microscopy: A Textbook for Materials Science. USA: Springer. p. 3–22

[17] Faraji M, Yamini Y, Rezaee M (2010). Magnetic nanoparticles*,* synthesis, stabilization, functionalization, characterization, and applications. J. Iran. Chem. Soc..

[18] Brar S, Verma M (2011). Measurement of nanoparticles by light-scattering techniques. TrAC, Trends Anal. Chem..

[19] Syed S, Chinthala P (2015). Heavy metal detoxification by different *Bacillus* species isolated from solar salterns. Scientifica.

[20] Lima de Silva, A.A., de Carvalho, M.A., de Souza, S.A., Dias, P.M., da Silva Filho, R.G., de Meirelles Saramago, C.S., de Melo Bento, C.A. & Hofer, E. (2012). Heavy metal tolerance (Cr, Ag and Hg) in bacteria isolated from sewage. *Braz. J. Microbiol.* 43(4), 1620–1631. https://europepmc.org/article/pmc/3769023 10.1590/S1517-838220120004000047 PMC3769023 24031994

[21] Zaki S, Kamal A, Elkady M, Abu-Elreesh G, Abd-El- Haleem D (2014). Biosynthesis of silver nanoparticles using *Stenotrophomonas rhizophila* and its application as a disinfectant agent of water. Eur. J. Exp..

[22] ELtarahony, M., Zaki, S., Kheiralla, Z. & Abd-El-Haleem, D. (2016). Biogenic synthesis of iron oxide nanoparticles via optimization of nitrate reductase enzyme using statistical experimental design. J. Adv. Biotechnol..

[23] Dawadi S, Katuwal S, Gupta A, Lamichhane U, Thapa R, Jaisi S, Lamichhane G, Bhattarai DP, Parajuli N (2021). Current research on silver nanoparticles: Synthesis, characterization, and applications. J. Nanomater..

[24] Tao F, Liu Y, Luo Q, Su F, Xu Y, Li F, Yu B, Ma C, Xu P (2011). Novel organic solvent-responsive expression vectors for biocatalysis: Application for development of an organic solvent-tolerant biodesulfurizing strain. Biores. Technol..

[25] Avendaño R, Chaves N, Fuentes P, Sánchez E, Jiménez JI, Chavarría M (2016). Production of selenium nanoparticles in *Pseudomonas putida* KT2440 OPEN. Sci. Rep..

[26] Tang H, Yu H, Tai C, Huang K, Liu Y, Wang L, Yao Y, Wu G, Xu P (2012). Genome sequence of a novel nicotine-degrading strain, *Pseudomonas geniculata* N1. J. Bacteriol..

[27] Laukova A, Kandričáková A, Ščerbová J, Strompfová V, Miltko R, Kowalik B, Belzecki G (2013). Properties of *Enterococcus thailandicus* isolates from beavers. Afr. J. Microbiol. Res..

[28] Ahmad T, Wani I, Al-Hartomy O, Al-Shihri A, Kalam A (2015). Low temperature chemical synthesis and comparative studies of silver oxide nanoparticles. J. Mol..

[29] Zhang J, Lan CQ (2008). Nickel and cobalt nanoparticles produced by laser ablation of solids in organic solution. Mater. Lett..

[30] Yuvakkumar R, Elango V, Rajendran V, Kannan N (2011). Preparation and characterization of zero valent iron nanoparticles. Dig. J. Nanomater. Biostruct..

[31] Annapurna S, Suresh Y, Sreedhar B, Bhikshamaiah G, Singh A (2014). Characterization of green synthesized copper nanoparticles stabilized by ocimum leaf extract. MRS Online Proc. Library Arch..

[32] Devasenan S, Beevi N, Jayanthi S (2016). Green synthesis and characterization of zinc nanoparticle using Andr*ographis paniculata* leaf extract. Int. J. Pharm. Sci. Rev. Res..

[33] Minaeian S, Shahverdi R, Nohi A, Shahverdi H (2008). Extracellular biosynthesis of silver nanoparticles by some bacteria. J. Sci. I. A. U (JSIAU).

[34] Shameli K, Bin AM, Emad A, Jaffar A, Ibrahim N, Shabanzadeh P, Rustaiyan A, Abdollahi Y, Bagheri S, Abdolmohammadi S, Usman M, Zidan M (2012). Green biosynthesis of silver nanoparticles using *Callicarpa maingayi* stem bark extraction. Molecules.

[35] Mishra A, Sardar M (2015). Cellulase assisted synthesis of nano silver and gold: Application as immobilization matrix for biocatalysis. Int. J. Biol. Macromol..

[36] Varshney R, Bhadauria S, Gaur M (2012). A review: Biological synthesis of silver and copper nanoparticles. Nano Biomed. Eng..

[37] Iravani S (2014). Bacteria in nanoparticle synthesis: Current status and future prospects. Int. Scholarly Res. Notices.

[38] Gomathy M (2010). Microbial mechanisms of heavy metal tolerance—A review. Agric. Rev..

[39] Deepak, V., Kalishwaralal, K., Pandian, S. & Gurunathan, S. (2011). An Insight into the Bacterial Biogenesis of Silver Nanoparticles, Industrial Production and Scale-up (chapter 2). In: Mahendra, R. & Nelson, D. (eds.). Metal Nanoparticles in Microbiology.1^st^ ed. Berlin: Springer Verlag.17–35.

[40] Omajali JB, Mikheenko IP, Merroun ML, Wood J, Macaskie LE (2015). Characterization of intracellular palladium nanoparticles synthesized by *Desulfovibrio desulfuricans* and *Bacillus benzeovorans*. J. Nanopart. Res..

[41] Markus J, Mathiyalagan R, Kim YJ, Abbai R, Singh P, Ahn S, Perez ZEJ, Hurh J, Yang DC (2016). Intracellular synthesis of gold nanoparticles with antioxidant activity by probiotic *Lactobacillus kimchicus* DCY51T isolated from Korean Kimchi. Enzyme Microb. Technol..

[42] Fatemi M, Mollania N, Momeni-Moghaddam M, Sadeghifar F (2018). Extracellular biosynthesis of magnetic iron oxide nanoparticles by *Bacillus cereus* strain HMH1: characterization and In vitro cytotoxicity analysis on MCF-7 and 3T3 Cell Lines. J. Biotechnol..

[43] Varshney R, Seema B, Gaur MS, Pasricha R (2010). Characterization of copper nanoparticles synthesized by a novel microbiological method. J. Miner. Metals Mater. Soc..

[44] Mashrai A, Uzzaman S (2014). Biological synthesis of ZnO nanoparticles using *C. albicans* and studying their catalytic performance in the synthesis of steroidal pyrazolines. Arab. J. Chem..

[45] Hachani R, Lowdell M, Birchall M, Hervault A, Mertz D, Begin-Colin S, Thanh NT (2016). Polyol synthesis, functionalisation, and biocompatibility studies of super-paramagnetic iron oxide nanoparticles as potential MRI contrast agents. Nanoscale.

[46] Deng D, Cheng Y, Jin Y, Qi T, Xiao F (2012). Antioxidative effect of lactic acid-stabilized copper nanoparticles prepared in aqueous solution. J. Mater. Chem..

[47] Gayduchenko I, Fedorov GE, Ibragimov RA, Stepanova T, Gazaliev A, Vysochanskiy N, Bobrov Y, Malovichko A, Sosnin I, Bobrinetskiy I (2015). Synthesis of single-walled carbon nanotube networks using monodisperse metallic nanocatalysts encapsulated in reverse micelles. Hemijska Industrija.

[48] Saliani M, Jalal R, Goharshadi EK (2015). Effects of pH and temperature on antibacterial activity of zinc Oxide nanofluid against *E. coli* O157:H7 and Staphylococcus aureus. Jundishapur J. Microbiol..

[49] Mohameed Q, Hattab F, Fakhry M (2015). Effect of substrate temperature on structural characteristics of nano silver oxide prepared by pulsed-laser deposition. Iraq. J. Appl. Phys..

[50] Dong H, Zhao F, Zeng G, Tang L, Fan C, Zhang L, Zeng Y, He Q, Xie Y, Wu Y (2016). Aging study on carboxymethyl cellulose-coated zero-valent iron nanoparticles (nZVI) in water: Chemical transformation and structural evolution. J. Hazard. Mater..

[51] Camacho-Flores BA, Martínez-Álvarez O, Arenas-Arrocena MC, García- Contreras R, Argueta-Figueroa L, De La Fuente-Hernández JY, Acosta-Torres LS (2015). Copper: Synthesis techniques in nanoscale and powerful application as an antimicrobial agent. J. Nanomater..

[52] Song AL, Yang W, Yang W, Sun G, Yin X, Gao L, Wang Y, Qin X (2017). Facile synthesis of cobalt nanoparticles entirely encapsulated in slim nitrogen-doped carbon nanotubes as oxygen reduction catalyst. ACS Sustain. Chem. Eng..

[53] Muniz-Miranda M, Gellini C, Giorgetti E (2011). Surface-enhanced raman scattering from copper nanoparticles obtained by laser ablation. J. Phys. Chem. C.

[54] Zamiri R, Rebelo A, Zamiri G, Adnani A, Kuashal A, Belsley MS, Ferreira J (2014). Far-infrared optical constants of ZnO and ZnO/Ag nanostructures. RSC Adv..

[55] Eltarahony M, Zaki S, Abd-El-Haleem D (2018). Concurrent synthesis of zero- and one-dimensional, spherical, rod-, needle-, and wire-shaped CuO nanoparticles by *Proteus mirabilis* 10B. J. Nanomater..

[56] Eltarahony M, Zaki S, ElKady M, Abd-El-Haleem D (2018). Biosynthesis, characterization of some combined nanoparticles, and its biocide potency against a broad spectrum of pathogens. J. Nanomater..

[57] Eivari AH, Rahdar A, Arabi H (2012). Preparation of super paramagnetic iron oxide nanoparticles and investigation their magnetic properties. Int. J. Sci. Eng. Investig..

[58] Margabandhu M, Sendhilnathan S, Senthilkumar S, Gajalakshmi D (2016). Investigation of structural, morphological, magnetic properties and biomedical applications of Cu^2+^ substituted uncoated cobalt ferrite nanoparticles. Braz. Arch. Biol. Technol..

[59] Raza MA, Kanwal Z, Riaz S, Naseem S (2016). Synthesis, characterization and antibacterial properties of nano-sized cobalt particles. Adv. Civ. Eng. Mater..

[60] Elilarassi R, Chandrasekaran G (2012). Synthesis, structural and magnetic characterization of Ni-Doped ZnO diluted magnetic semiconductor. J. Mater. Sci..

[61] Ahmad E, Zaidi A, Khan MS, Oves M, Zaidi A (2012). Heavy metal toxicity to symbiotic nitrogen-fixing microorganism and host legumes. Toxicity of heavy metals to legumes and bioremediation.

[62] Rout A, Jena P, Sahoo D, Bindhani B (2014). Green synthesis of silver nanoparticles of different shapes and its antibacterial activity against *Escherichia coli*. Int. J. Curr. Microbiol. Appl. Sci..

[63] Lee C, Kim JY, Lee WI, Nelson KL, Yoon J, Sedlak DL (2008). Bactericidal effect of zero-valent iron nanoparticles on *Escherichia coli*. Environ. Sci. Technol..

[64] Tran N, Mir A, Mallik D, Sinha A, Nayar S, Webster TJ (2010). Bactericidal effect of iron oxide nanoparticles on *Staphylococcus aureus*. Int. J. Nanomed..

[65] Arakha M, Saleem M, Mallick BC, Jha S (2015). The effects of interfacial potential on antimicrobial propensity of ZnO nanoparticle. Sci. Rep..

[66] Stoimenov PK, Klinger RL, Marchin GL, Klabunde KJ (2002). Metal oxide nanoparticles as bactericidal agents. Langmuir.

[67] Sohm B, Immel F, Bauda P, Pagnout C (2015). Insight into the primary mode of action of TiO_2_ nanoparticles on *Escherichia coli* in the dark. Proteomics.

[68] Zhang L, Jiang Y, Ding Y, Povey M, York D (2007). Investigation into the antibacterial behaviour of suspensions of ZnO nanoparticles (ZnO nanofluids). J. Nanoparticle Res..

[69] Huh AJ, Kwon YJ (2011). Nanoantibiotics: A new paradigm for treating infectious diseases using nanomaterials in the antibiotics resistant era. J Control Release..

[70] Chatterjee AK, Chakraborty R, Basu T (2014). Mechanism of antibacterial activity of copper nanoparticles. Nanotechnology.

[71] Khalil AT, Ovais M, Ullah I, Ali M, Shinwari ZK, Maaza M (2017). Physical properties, biological applications and biocompatibility studies on biosynthesized single phase cobalt oxide (Co3O4) nanoparticles via Sageretia thea (Osbeck). Arab. J. Chem..

[72] Niemirowicz K, Piktel E, Wilczewska AZ, Markiewicz KH, Durnaś B, Wątek M, Puszkarz I, Wróblewska M, Niklińska W, Savage PB (2016). Core–shell magnetic nanoparticles display synergistic antibacterial effects against *Pseudomonas aeruginosa* and *Staphylococcus aureus* when combined with cathelicidin LL-37 or selected ceragenins. Int. J. Nanomed..

[73] Ruparelia JP, Chatterjee AK, Duttagupta SP, Mukherji S (2008). Strain specificity in antimicrobial activity of silver and copper nanoparticles. Acta Biomater..

[74] Aysa NH, Salman HD (2016). Antibacterial activity of modified zinc oxide nanoparticles against *Pseudomonas aeruginosa* isolates of burn infections. World Sci. News.

[75] American Public Health Association (APHA) (1995). Standard Methods for Estimation of Water and Wastewater.

[76] Naziri D, Hamidi M, Hassanzadeh S, Tarhriz V, Zanjani BM, Nazemyieh H, Hejazi MA, Hejazi MS (2014). Analysis of carotenoid production by *Halorubrum* sp. TBZ126, an extremely halophilic archeon from Urmia Lake. Adv. Pharmac. Bull..

[77] Bailey R, Scott EG (1966). Diagnostic Microbiology: a Textbook for the Isolation and Identification of Pathogenic Microorganisms.

[78] Antonio L, Jesu D, Sabah E, Conrado M, Jose M, Ferna N (2002). Nitrate is reduced by heterotrophic bacteria but not transferred to *Prochlorococcus* in non-axenic cultures. FEMS Microbiol. Ecol..

[79] Staley JT, Boon DR, Garrity GM, Devos P, Fellow MG, Rainey FA, Schlifer KH, Brenner DJ, Castenholz RW, Holt JG, Krieg NR, Liston J, Moulder JW, Murray RG, Niven CF, Pfenning N, Sneath PH, Jully JG, Williams S (2005). Bergey’s Manual of Systematic Bacteriology.

[80] Prescott LM, Harley JP, Klein DA (2005). Microbiology.

[81] DebRoy S, Das L, Ghosh S, Banerjee S (2012). Isolation of nitrate and phosphate removing bacteria from various environmental sites. OnLine J. Biol. Sci..

[82] Abd-El-Haleem, D. (2005). DNA extraction kit from bacteria, fungi, waters and whole blood, [Egyptian patent office ASRT. Regist. Patent. No. (223525)].

[83] Wang P, Li X, Xiang M, Zhai Q (2007). Characterization of efficient aerobic denitrifiers isolated from two different sequencing batch reactors by 16S-rDNA analysis. J. Biosci. Bioeng..

[84] Elrashdy R, Abd-El-Haleem D (2005). Molecular analysis of cross-bacterial contamination detected during diagnosis HCV infection. J. Appl. Sci. Environ. Manag..

[85] Tamura K, Stecher G, Peterson D, Filipski A, Kumar S (2013). MEGA 6*,* molecular evolutionary genetics analysis, version 6.0. Mol. Biol. Evol..

[86] Zaki S, Elkady M, Farag S, Abd-EL-Haleem D (2012). Determination of the effective origin source for nanosilver particles produced by *Escherichia coli* strain S78 and its application as antimicrobial agent. Mater. Res. Bull..

[87] Kamal A, Zaki S, Abu-Elreesh G, Abd-El-Haleem D (2016). Biosynthesis and characterization of silver nanoparticles using *Metschnikowia pulcherrima* strain 29a*,* their antibacterial, antifungal and bioluminescent toxicity effects against microbial pathogens. Ecol. Environ. Conserv..

[88] Rai M, Duran N (2011). Metal Nanoparticles in Microbiology.

[89] Park MJ, Gwak KS, Yang I, Kim KW, Jeung EB, Chang JW (2009). Effect of citral, eugenol, nerolidol and α-terpineol on the ultrastructural changes of *Trichophyton mentagrophytes*. Fitoterapia.

[90] Crespi J, Quici N, Halac EB, Leyva AG, Ramos CP, Mizrahi M, Requejo FG, Litter MI (2016). Removal of uranium (VI) with iron nanoparticles. Chem. Eng. Trans..

[91] Rahman MM, Khan SB, Jamal A, Faisal M, Aisiri AM (2011). Iron oxide nanoparticles. Nanomaterials.

[92] Michalak I, Chojnacka K, Marycz K (2011). Using ICP-OES and SEM-EDX in biosorption studies. Mikrochim. Acta.

[93] Arokiyaraj S, Saravanan M, Udaya N, Prakash K, Valan Arasu M, Vijayakumar B, Vincent S (2013). Enhanced antibacterial activity of iron oxide magnetic nanoparticles treated with *Argemone mexicana* L. leaf extract*,* an in vitro study. Mater. Res. Bull..

[94] Avadi MR, Sadeghi AM, Tahzibi A, Bayati K, Pouladzadeh M, Zohuriaan-Mehr MJ (2004). Diethylmethyl chitosan as an antimicrobial agent*,* synthesis, characterization and antibacterial effects. Eur. Polymer J..

[95] Shoeib AA, Alkufeidy RMS (2014). Bacteriostatic or bactericidal action of four aqueous plant extracts on multi-drug resistant bacteremia and their effect on cells morphology recorded using scanning electron microscopy (SEM). Afr. J. Microbiol. Res..

[96] Ashmawy NA, El-Bebany AF, Shams A (2020). Identification and differentiation of soft rot and blackleg bacteria from potato using nested and multiplex PCR. J. Plant Dis. Protect..

[97] Fréchon D, Exbrayat P, Helias V, Hyman LJ, Jouan B, Llop P, Lopez MM, Payet N, Perombelon MCM, Toth IK, Van Backhoven JRCM, Van der Wolf JM, Berthaeu Y (1998). Evaluation of a PCR kit for the detection of *Erwinia carotovora* subsp. *atroseptica* on potato tubers. Potato Res..

[98] Shoeib AA, Ashmawy NA, Hammad S, Hafez E (2016). Molecular and biological identification of *Erwinia amylovora* Egyptian isolates compared with other German strains. J. Plant Physiol. Pathol..

[99] Younis AM, Shoeib AA, Elsaedy MA, Osman KA (2016). Efficacy of ozone and hydrogen peroxide on controlling crown gall bacterium and root knot nematode infected Guava plants in Egypt. Alexandr. J. Agric. Sci..

