# Supplementary material for: Nano-metals forming bacteria in Egypt. I. Synthesis, characterization and effect on some phytopathogenic bacteria in vitro
Source: Sci Rep. 2021 Jun 18;11:12876. doi: 10.1038/s41598-021-92171-6 (PMC8213709; doi:10.1038/s41598-021-92171-6)
Supplement: Supplementary file 1 — Supplementary Information. [file 41598_2021_92171_MOESM1_ESM.docx]

**Ref: Submission ID 4549dc4c-93be-471c-8c6d-a8f617175332**

30^th^ April 2021

**Supplementary**

| **Isolate Code**  **Tests** | **E12** | **M69** | **T65** | **G80** |
| --- | --- | --- | --- | --- |
| **Morphological and Physiological tests:** | | | | |
| Colony | cream | white | withe | white |
| Pigment production | -* | - | - | - |
| Shape | COCCI | Rod | Rod | Rod |
| Gram stain | G+ ** | G- | G- | G- |
| pH 9 | **+** | **+** | **+** | **+** |
| pH 3 | **-** | **-** | **-** | **-** |
| 5% NaCl | **+** | **+** | **-** | **+** |
| Growth at 40 °C | **-** | **+** | **-** | **+** |
| Growth at 25°C | **+** | **+** | **+** | **+** |
| Growth at 4°C | **-** | **+** | **+** | **-** |
| Motility | **-** | **+** | **+** | **+** |
| Spore formation | **-** | **-** | **-** | **-** |
| Anaerobic growth | **+** | **-** | **+** | **+** |
| **Biochemical tests:** | | | | |
| Oxidase | - | + | + | + |
| Catalase | - | + | + | + |
| Starch | - | - | - | - |
| Blood Agar | - | - | + | + |
| H_2_S | - | - | - | - |
| Lysin decarboxylase | - | - | - | + |
| lipase | - | - | + | + |
| Esterase | - | - | + | + |
| urease | - | + | - | + |
| alkaline phosphatase | + | **-** | **-** | **-** |
| DNAase | - | - | + | + |
| Voges–Proskauer | - | - | - | - |
| Indol | - | - | - | + |
| Gelatin liquefaction | + | - | - | + |
| O-nitrophenyl-beta-D-galactopyranoside (ONPG) | **-** | **-** | **-** | **-** |
| Arginine | **+** | **-** | **+** | **+** |
| beta-glucuronidase | **+** | **-** | **-** | **+** |
| Phenylealanine | **-** | **-** | **-** | **-** |
| N-acetyl-glucosaminide | **-** | **+** | **-** | **-** |
| Malonate | **-** | **+** | **-** | **-** |
| Gamma-glutamyl transferase | **-** | **-** | **+** | **-** |
| beta-glucosidase | **-** | **+** | **-** | **-** |
| beta-galactosidase | **-** | **+** | **-** | **-** |
| Esculine | **-** | **+** | **-** | **-** |
| Nitrates | **+** | **+** | **-** | **-** |
| **Sugars fermentation:** | | | | |
| Sorbitol | **-** | **-** | **-** | **-** |
| Mannitol | **-** | **-** | **-** | **-** |
| Fructose | **+** | **+** | **-** | **+** |
| glucose | **+** | **+** | **+** | **+** |
| galactose | **+** | **-** | **-** | **+** |
| ribose | **-** | **-** | **-** | **-** |
| Sucrose | **+** | **-** | **-** | **-** |
| Maltose | **+** | **-** | **-** | **-** |
| Citrate | **-** | **+** | **+** | **-** |
| Sorbitol | **-** | **-** | **-** | **+** |
| Trehalose | **-** | **-** | **-** | **-** |
| Cellobiose | **-** | **-** | **-** | **-** |
| lactose | **-** | **-** | **-** | **-** |
| malonate | **-** | **+** | **-** | **+** |
| Dulcitol | **-** | **+** | **-** | **-** |
| Adonitol | **-** | **-** | **-** | **-** |
| Rhamnose | **-** | **+** | **-** | **-** |
| Raffinose | **-** | **-** | **-** | **-** |
| Inositol | **-** | **-** | **-** | **-** |

*+: test positive, -: test negative, **G-: Gram negative, G+: Gram positive

**S1. Morphological, physiological, biochemical and sugar fermentation tests of the isolated NPs forming bacteria.** …………………………………………………………….………………………………………………

| **Isolate Code** | **Genus/ species** | **Accession No** |
| --- | --- | --- |
| **E 12** | *Enterococcus thailandicus* | MG831199 |
| **M 69** | *Marinobacter hydrocarbonoclasticus* | MG833231 |
| **T 65** | *Pseudomonas putida* | MG833008 |
| **G 80** | *Pseudomonas geniculata* | MG831721 |

**S2. Isolate code and Gene Bank Accession No. of the NPs forming bacteria identified**

……………………………………………………………………………………………….………………………………………………………


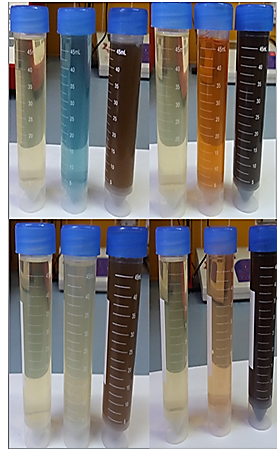


**D**

**C**

**B**

**A**

Fe-NPS

LB + Cu salt

LB Medium

LB +Fe salt

L.B Medium

Cu-NPS

Co-NPS

LB +Co salt

LB Medium

Zn-NPS

LB +Zn salt

LB Medium

**S3. Production of metallic NPs: (A)** Cu**, (B)** Fe**, (C)** Zn **and (A)** Co, **in comparison**

**with Luria Bertani (LB) medium free of metals and LB + metal salt at Zero time (from left to right).**

………………………………………………………………………………………………………………………………………

| 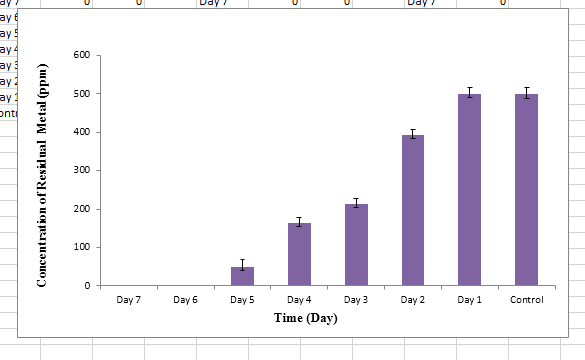  **CuNPs**  **B** | 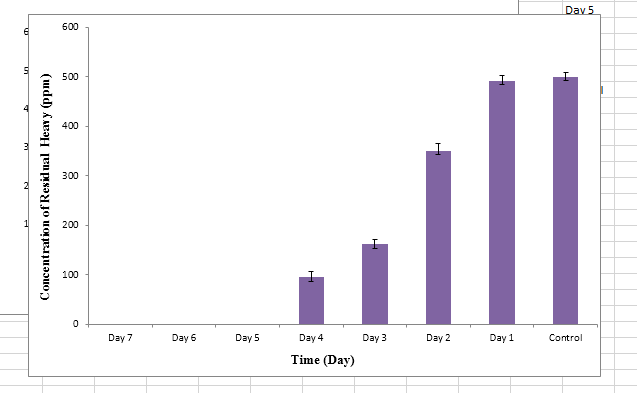  **FeNPs**  **A** |
| --- | --- |
| 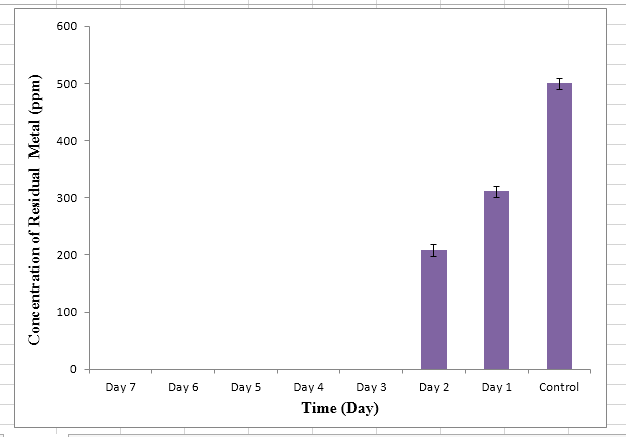  **D**  **ZnNPs** | 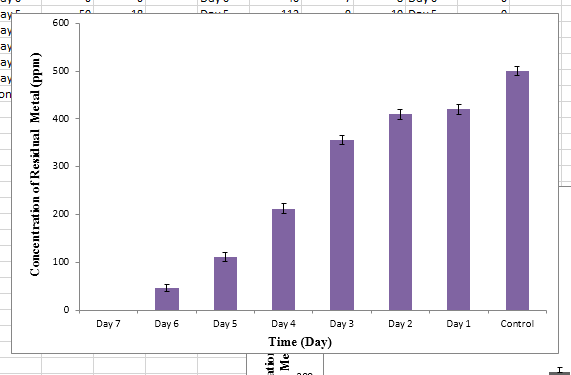  **CoNPs**  **C** |

**S4. Metal concentration in the cell-free supernatant was determined by Inductively Coupled Plasma-Optical Emission Spectroscopy. Residual of FeNPs (A), CuNPs (B), CoNPs (C) and ZnNPs (D).**

………………………………………………………………………………………………………………………………………


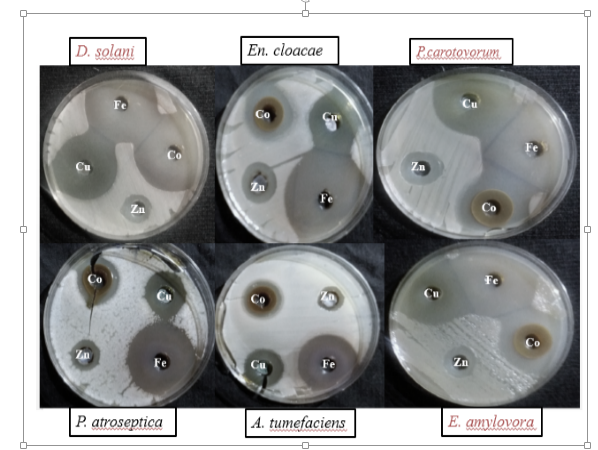


*P. atrosepticum*

*E. amylovora*

*A . tumefaciens*

*E. cloacae*

*P. carotovorum* subsp. *carotovorum.*

*D. solani*

**S5. Antibacterial activity of Fe**, **Cu**, **Co** and **Zn** Nanoparticles (**NPs) on the growth of some phytopathogenic bacteria**

………………………………………………………………………………………………………………………………………………………
